# Specification of jaw identity by the Hand2 transcription factor

**DOI:** 10.1038/srep28405

**Published:** 2016-06-22

**Authors:** Noriko Funato, Hiroki Kokubo, Masataka Nakamura, Hiromi Yanagisawa, Yumiko Saga

**Affiliations:** 1 Research Center for Medical and Dental Sciences, Tokyo Medical and Dental University (TMDU), 1-5-45 Yushima, Bunkyo-ku, Tokyo 113-8510, Japan; 2 Division of Mammalian Development, National Institute of Genetics, Yata 1111, Mishima, Shizuoka 411-8540, Japan; 3 Department of Genetics, The Graduate University for Advanced Studies, Yata 1111, Mishima, Shizuoka 411-8540, Japan; 4 Department of Cardiovascular Physiology and Medicine, Graduate School of Biomedical and Health Sciences, Hiroshima University, 1-2-3 Kasumi, Minamiku, Hiroshima 734-8551, Japan; 5 Department of Molecular Biology, University of Texas Southwestern Medical Center, 5323 Harry Hines Blvd., Dallas, TX, 75390-9148, USA; 6 Life Science Center of Tsukuba Advanced Research Alliance, University of Tsukuba, Tennodai 1-1-1, Tsukuba, Ibaraki 305-8577, Japan

## Abstract

Acquisition of the lower jaw (mandible) was evolutionarily important for jawed vertebrates. In humans, syndromic craniofacial malformations often accompany jaw anomalies. The basic helix-loop-helix transcription factor Hand2, which is conserved among jawed vertebrates, is expressed in the neural crest in the mandibular process but not in the maxillary process of the first branchial arch. Here, we provide evidence that Hand2 is sufficient for upper jaw (maxilla)-to-mandible transformation by regulating the expression of homeobox transcription factors in mice. Altered *Hand2* expression in the neural crest transformed the maxillae into mandibles with duplicated Meckel’s cartilage, which resulted in an absence of the secondary palate. In *Hand2-*overexpressing mutants, non-Hox homeobox transcription factors were dysregulated. These results suggest that Hand2 regulates mandibular development through downstream genes of Hand2 and is therefore a major determinant of jaw identity. Hand2 may have influenced the evolutionary acquisition of the mandible and secondary palate.

The evolution of vertebrates, which first appeared as gnathostomes (jawed vertebrates), is associated with jaw acquisition, as it allowed a shift from passive to active predation^1^. The evolution of articulated jaws was mainly induced because invertebrates acquired the neural crest^2^. The upper (maxilla) and lower (mandible) jaws and associated soft tissues are derived from the maxillary and mandibular processes, respectively, of the first branchial arch. The jaws of early gnathostomes consisted of two major components, the palatoquadrate in the upper jaw and Meckel’s cartilage in the lower jaw^3^,^4^. Jawed vertebrates are divided into two groups, bony vertebrates and cartilaginous fishes, which are further grouped into two lineages, elasmobranchs (sharks and rays) and holocephalans (elephant sharks and ratfishes). Holocephalans, which are the most primitive of living jawed vertebrates^5^, have a complete hyoid arch and upper jaw fused to the cranium. Cyclostomes (lampreys) are widely recognised as the closest living relatives of jawless vertebrates^1^,^6^,^7^. In humans, improper development of neural crest cells (NCCs) occurs in at least one-third of all congenital anomalies^8^. Syndromic craniofacial malformations often accompany mandibular anomalies such as agnathia, micrognathia, retrognathia, and aplasia/hypoplasia of the mandibular condyles. Therefore, the identification of factors involved in mandible specification will provide insights into the evolution of vertebrates and the mechanisms underlying congenital anomalies involving craniofacial tissues.

The Hand protein family of basic helix-loop-helix (bHLH) transcription factors in higher species consists of two members, Hand1 and Hand2. Ablation of *Hand2* expression in NCCs of the branchial arches results in hypomorphism of the mandibles and Meckel’s cartilage in mice^9^,^10^,^11^. The NCCs in the first branchial arch are devoid of *Hox* homeobox genes, and *Hox* gene expression in the first branchial NCCs is incompatible with jaw formation^12^. Instead, non-Hox homeobox transcription factors, which are expressed in the first branchial arch, have broad potential to produce most of the cartilaginous and dermatocranial derivatives^13^. Dlx homeobox transcription factors, which specify the dorsoventral patterning of the first branchial arch, have been involved in vertebrate evolution^4^,^14^. Dlx6 acts as an intermediary between endothelin1 (*Edn1*)/endothelin receptor type A (*Ednra*)-mediated signalling and *Hand2* expression during mandibular development^15^,^16^, although *Hand2* expression in the ventral region of the branchial arch is independent of *Edn1/Ednra*-mediated signals^9^. The loss of *Dlx5/Dlx6* or *Ednra* in mice results in homeotic transformation of the lower jaws to the upper jaws^4^,^17^,^18^, whereas the constitutive activation of *Ednra* expression induces ectopic expression of *Hand2* in the maxillary arch and subsequently transforms the upper jaws into lower jaws^19^,^20^. In 66% of *Ednra*^*Hand2/*+^ mutants, in which one copy of *Hand2* was knocked into the *Ednra* locus, exhibit the transformation of upper jaw structures into lower jaw elements^19^. In the Japanese lamprey *Lethenteron japonicum*, a *Hox*-expression-free mandible has been maintained^21^ and homologs of *Edn1, Ednra*, and *Hand2* are expressed as in gnathostomes^22^, thus indicating that the regulation of *Hand2* gene expression in the branchial arch is conserved in cyclostomes and gnathostomes.

Here, we show that altered expression of the Hand transcription factors, Hand1 and Hand2, in NCCs or the osteochondral progenitors induced the transformation of the upper jaw into a lower jaw, which resulted in a missing secondary palate. We identified that Hand2 was specifically located genetically upstream of non-Hox homeobox transcription factors in the development of the first branchial arch. Modification of the *Hand2* gene may correlate with the evolution of vertebrates.

## Results

### Altered expression of *Hand2* induces the transformation of the upper jaw to the lower jaw

**Figure 1:**
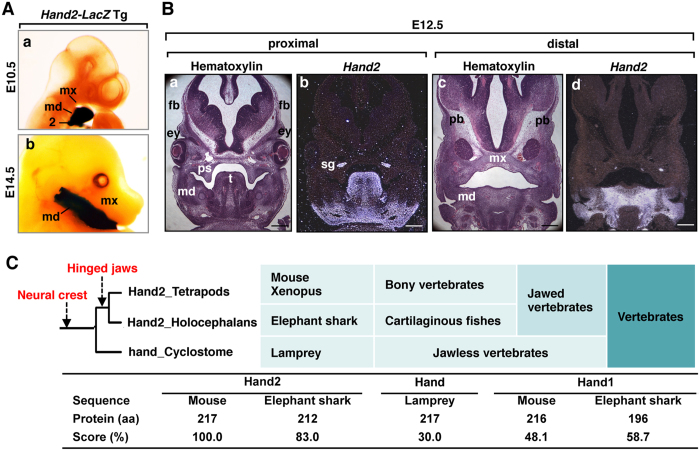
Nested expression of *Hand2* in the mandibular arch. (**A**) Whole-mount β-galactosidase staining of *Hand2-LacZ* transgenic embryos at embryonic day (E) 10.5 (a) and E14.5 (b). md, mandibular arch; mx, maxillary arch; 2, the second branchial arch. (**B**) *Hand2* (b,d) was detected by *in situ* hybridization on coronal sections from the wild-type head at E12.5. Adjacent control sections are stained with hematoxylin (a,c). *Hand2* expression is nested in the mandibular process (md). mx, maxillary process; fb, frontal bone; ps, palatal shelf; t, tongue; pb, parietal bone; sg, sympathetic ganglion. Scale bars: 300 μm. (**C**) Genetic events and phylogeny of the Hand2 coding sequences.

The bHLH transcription factor Hand2 controls cardiac, limb, and craniofacial development^9^,^11^,^15^,^23^,^24^. When we analysed the whole-mount LacZ expression that was driven by the 11-kb *Hand2* promoter in the craniofacial region, *Hand2* expression was observed exclusively in the mandibular arch but not in the maxillary arch or calvarial regions (Fig. 1A). The nested expression of the *Hand2* gene in the mandibular region was also confirmed by *in situ* hybridization (Fig. 1B). Because the acquisition of the mandible was especially important in the evolution of vertebrates and *Hand2* expression is nested in the mandibular arch, the amino-acid alignment of the Hand proteins was examined. Interestingly, Hand2 is evolutionally conserved in primitive jawed vertebrates (elephant sharks) but less conserved among jawless vertebrates (lampreys) and invertebrates (Fig. 1C; Supplementary Figs S1 and S2).

**Figure 2:**
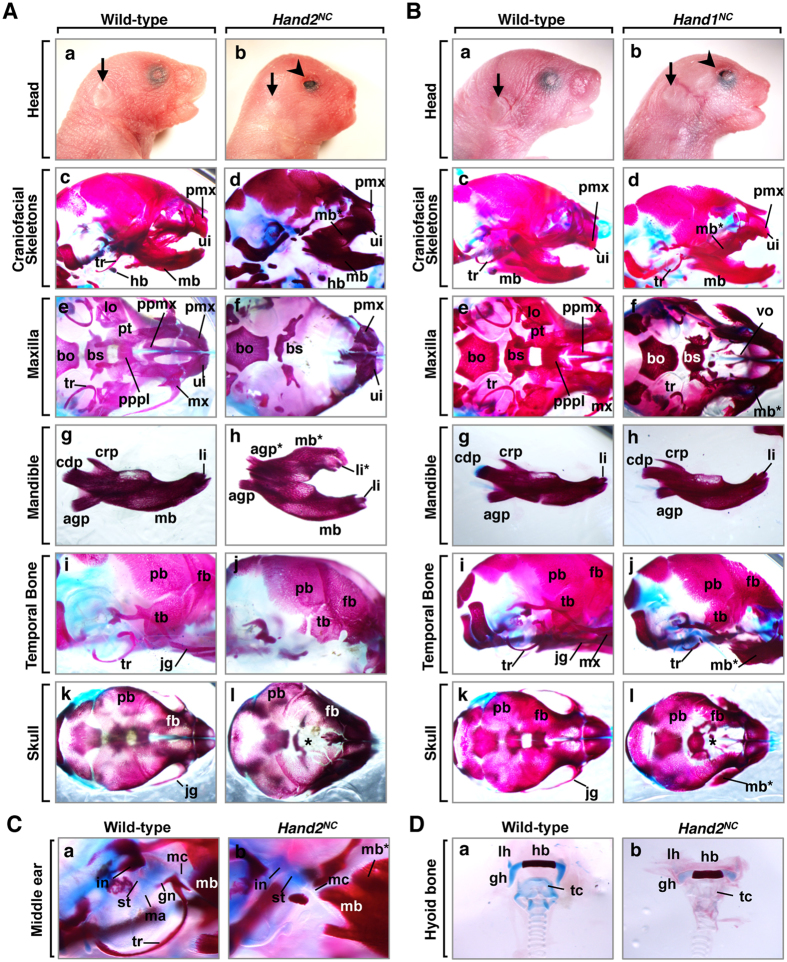
Patterning defects in neural crest-specific *Hand2*- and *Hand1-*induced mutants. (**A**) Phenotypic analysis of *Hand2*^*CAT/*+^*; Wnt1-Cre* (*Hand2*^*NC*^) mice. (a,b) Facial appearance of postnatal day (P) 1 wild-type (a) and *Hand2*^*NC*^ (b) mice. *Hand2*^*NC*^ mutants (b) exhibit brachycephaly, eyelid colobomas (arrowhead in b), and small pinna (arrow in b). (c–l) Alizarin red (mineralised bone) and alcian blue (cartilage) staining of P1 wild-type (c,e,g,i,k) and *Hand2*^*NC*^ (d,f,h,j,l) mice. *Hand2*^*NC*^ mutants demonstrate the transformation of the maxilla (mx) into a duplicated mandibular bone (mb*), which appears as a mirror image of the mandibular bone (mb). Hypoplasia of the frontal bones (fb) and frontal foramina (asterisk) are observed in the skull vault of *Hand2*^*NC*^ mutants (l). (**B**) Phenotypic analysis of *Hand1*^*CAT/*+^*; Wnt1-Cre* (*Hand1*^*NC*^) mice. (a,b) Facial appearance of P1 wild-type (a) and *Hand1*^*NC*^ (b) mice. *Hand1*^*NC*^ mutants (b) exhibit brachycephaly, eyelid colobomas (arrowhead in b), and small pinna (arrow in b). (c–l) Bone staining of P1 wild-type (c,e,g,i,k) and *Hand1*^*NC*^ (d,f,h,j,l) mice. *Hand1*^*NC*^ mutants have a partially duplicated mandible (mb*). Hypoplasia of the frontal bones (fb) and frontal foramina (asterisk) are observed in the skull vault of *Hand1*^*NC*^ mutants (l). (**C**) The middle ear phenotype of P1 wild-type (a) and *Hand2*^*NC*^ (b) mice. In wild-type mice (a), the malleus (ma), incus (in), stapes (st), gonial bone (gn), tympanic ring (tr), and Meckel’s cartilage (mc) are observed. In *Hand2*^*NC*^ mutants, the malleus, gonial bone, and tympanic ring are absent or hypomorphic. (**D**) The hyoid bone phenotype of P1 wild-type (a) and *Hand2*^*NC*^ (b) mice. The ossification of the hyoid bones is hypoplastic in *Hand2*^*NC*^ mutants. mx, maxilla; mb*, duplicated mandibular bone; mb, mandibular bone; fb, frontal bone; tr, tympanic ring; lo, lamina obturans; jg, jugal bones; tb, temporal bone; bs, basisphenoid bone; ui, upper incisors; li, lower incisors; li*, duplicated lower incisors; bo, basioccipital bone; pmx, premaxilla; hb, hyoid bone; pt, pterygoid; ppmx, palatal process of maxilla; pppl, palatal process of palatine; agp, angular process; crp, coronoid process; cdp, condylar process; pb, parietal bone; vo, vomer; hy, hyoid bone, with lesser (lh) and greater (gh) horns; tc, thyroid cartilage.

The correlation of the pattern of the nested expression of *Hand2* in the mandibular arch (Fig. 1A,B) and the phylogenetic tree of Hand2 (Fig. 1C; Supplementary Fig. S1) suggested that Hand2 might determine the mandibular identity within the first branchial arch. NCCs, which contribute to jaw and craniofacial evolution, have been implicated in the patterning of craniofacial cartilaginous and dermatocranial derivatives^25^. To explore the hypothesis that Hand2 specifies the identity of jaws within the NCCs of the branchial arches, we overexpressed *Hand2* specifically in the NCCs of mice and examined the resulting craniofacial phenotypes. At postnatal day (P) 1, all (16 of 16) *Hand2*^*CAT/*+^*; Wnt1-Cre* (hereafter *Hand2*^*NC*^) mice died neonatally with craniofacial deformities, including brachycephaly, eyelid colobomas, tongue protrusion, and small pinna in the anterior position, compared to control littermates (Fig. 2Ab; Supplementary Table S1). Alizarin red and alcian blue staining showed that the maxillary elements (maxillary bone, palatal process of maxilla and palatine, vomer, pterygoid, lamina obturans, and jugal bones) were aplastic in *Hand2*^*NC*^ mutants (Fig. 2Af; Supplementary Table S2) and that these elements were replaced by mirror-image duplication of the mandibular bone with duplicated lower incisors and mandibular molar alveoli (Fig. 2Ad,f,h). The mutant mandibular bones and angular processes of the mandible were hypoplastic, and the coronoid and condylar processes were aplastic (Fig. 2Ah). The duplicated mandibular bone was fused to the original mandibular bone at the condylar process and attached to the premaxilla (Fig. 2Ad,h). The premaxilla, which is a derivative of the frontonasal process (FNP), was mildly deformed, while the upper incisors within the premaxilla were maintained in *Hand2*^*NC*^ mutants (Fig. 2Af). The pterygoid, presphenoid, and basisphenoid bones, which are derivatives of the neural crest, were aplastic or severely malformed, whereas the basioccipital bone, which is a derivative of the mesoderm, was intact (Fig. 2Af). The temporal bones were reduced and fragmented in *Hand2*^*NC*^ mutants (Fig. 2Aj). Compared with the wild-type mice, the neural crest-derived frontal bones were much reduced in size in *Hand2*^*NC*^ mutants, whereas the mesoderm-derived parietal bones were similar in size (Fig. 2Ak,l). The mutant skulls showed brachycephaly (Fig. 2Ak,l), suggesting that skull shape was sensitive to the patterning information from the tissue at the junction of the maxillary process. In the middle ear, derivatives of the first branchial arch (malleus, incus, gonial bone, and tympanic ring) failed to form (Fig. 2C). The hyoid bones, which are derivatives of the second branchial arch, were maintained; however, the ossification was hypoplastic in *Hand2*^*NC*^ mutants (Fig. 2D).

**Figure 3:**
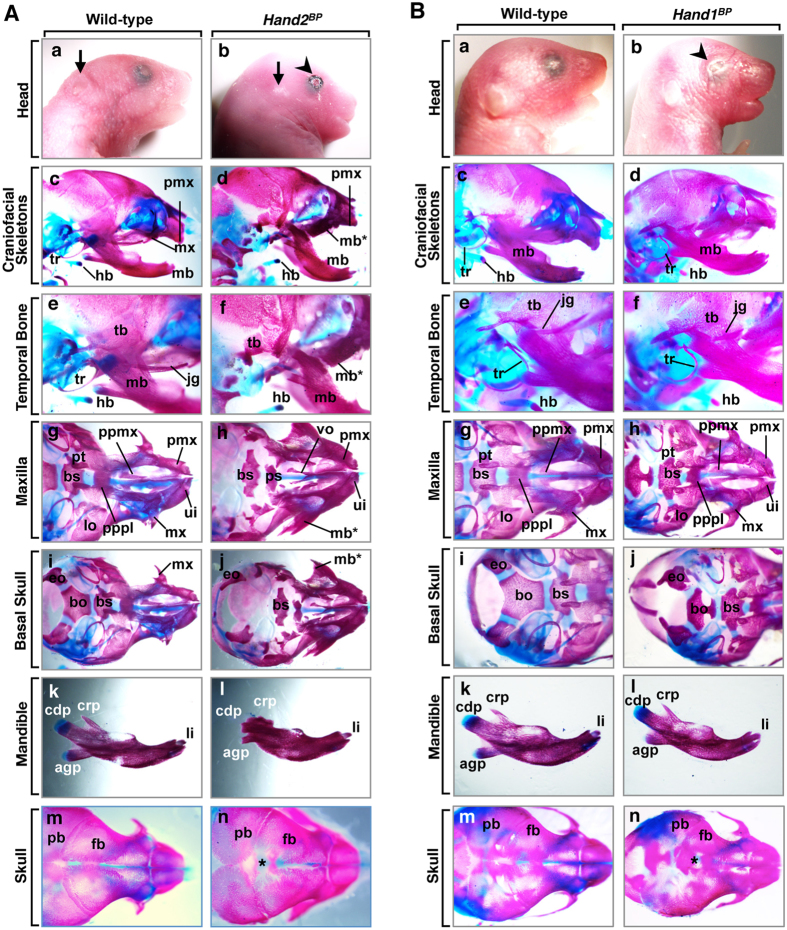
Craniofacial dysmorphism resulting from conditional activation of *Hand2* or *Hand1* by *Twist2-Cre.* (**A**) Phenotypic analysis of *Hand2*^*BP*^ mice. (a,b) Facial appearance of P1 wild-type (a) and *Hand2*^*BP*^ (b) mice. The *Hand2*^*BP*^ mutant exhibits brachycephaly, eyelid colobomas (arrowhead), and small pinna (arrow) (b). (c–n) Alizarin red and alcian blue staining of P1 wild-type and *Hand2*^*BP*^ mice, as indicated. *Hand2*^*BP*^ mutants show partial maxilla-to-mandible transformation (d,f,h,j). The tympanic ring (tr), jugal bone (jg), and basioccipital bone (bo) are aplastic in *Hand2*^*BP*^ mutants (f,j). The condylar processes and temporal bone (tb) of *Hand2*^*BP*^ mutant are severely hypoplastic (l). Hypoplasia of the frontal bones (fb) and open frontal foramina (asterisk) are observed in the skull vault of *Hand2*^*BP*^ mutants (n). (**B**) Phenotypic analysis of *Hand1*^*BP*^ mice. (a,b) Facial appearance of the P1 wild-type (a) and *Hand1*^*BP*^ (b) mice. *Hand1*^*BP*^ mutant (b) exhibits brachycephaly and eyelid colobomas (an arrowhead). (c-n) Alizarin red and alcian blue staining of P1 wild-type and *Hand1*^*BP*^ mice, as indicated. The pterygoid bone (pt), basisphenoid bone (bs), and basioccipital bone (bo) of *Hand1*^*BP*^ mutants are hypoplastic (h,j). Hypoplasia of the frontal bones (fb) and open frontal foramina (asterisk) are observed in the skull vault of *Hand1*^*BP*^ mutant (n). li, lower incisor; ui, upper incisor; pmx, premaxilla; pt, pterygoid; lo, lamina obturans; ppmx, palatal process of maxilla; pppl, palatal process of palatine; vo, vomer; eo, exoccipital bone; agp, angular process; crp, coronoid process; cdp, condylar process; pb, parietal bone.

To understand the physiological role of Hand2 in the bone development of the branchial arches, we overexpressed *Hand2* in the early precursors of all of the cell types of the bone primordium with *Twist2-Cre* knockin mice^26^. All *Hand2*^*CAT/*+^*; Twist2-Cre* (hereafter *Hand2*^*BP*^) mice died neonatally with severe craniofacial defects, including brachycephaly, eyelid colobomas, and small pinna (Fig. 3A), which were similar to the defects observed in the *Hand2*^*NC*^ mutants (Fig. 2A). In *Hand2*^*BP*^ mutants, the maxillary structures (the palatal process of the maxilla and palatine jugal bones) were missing and replaced by a partially duplicated set of mandibular bones with a mandibular molar alveolus (Fig. 3A). These skeletal abnormalities of *Hand2*^*BP*^ mutants were similar to but milder than those seen in *Hand2*^*NC*^ mutants (Fig. 2A), except for the basioccipital bones (Fig. 3A; Supplementary Table S2). These results indicate that *Hand2* in the NCCs contribute as a selector gene to the specification of the mandibular bone. The abnormalities were not observed in *Hand2*^*CAT/*+^ mice (n = 16).

### Altered expression of *Hand1* partially recapitulates *Hand2*-induced skeletal patterning

We next examined whether the neural crest phenotype was specific for the Hand protein family. To test whether Hand1 also transforms the upper jaws to the lower jaws, we used *Hand1*^*CAT/*+^*; Wnt1-Cre* (hereafter *Hand1*^*NC*^) mice and examined the craniofacial phenotypes at P1. *Hand1*^*NC*^ mutants resembled *Hand2*^*NC*^ mutants with their brachycephaly and eyelid colobomas (Fig. 2Bb). Bone analysis of *Hand1*^*NC*^ mutants revealed that the maxillary structures were hypoplastic and were replaced by a partially duplicated set of mandibular bones with mandibular molar alveoli (Fig. 2Bd,f; Supplementary Table S2). When we overexpressed *Hand1* specifically in the osteochondral progenitors of the bone primordium, *Hand1*^*CAT/*+^*; Twist2-Cre* (hereafter *Hand1*^*BP*^) mutants showed similar but weak phenotypes (Fig. 3B) compared with *Hand1*^*NC*^ mutants (Fig. 2B). Abnormalities were not observed in *Hand1*^*CAT/*+^ mice (n = 16).

The significance of the endoderm- and ectoderm-derived epithelium for craniofacial development has been demonstrated^27^,^28^. To examine the contribution of Hand transcription factors to the epithelial lineage in jaw development, we crossed *Hand2* or *Hand1* transgenic mice with the *KRT14-Cre* mice and examined the craniofacial phenotypes. The altered expression of the conditional alleles for *Hand2* or *Hand1* in the *KRT14-Cre* mice resulted in a lack of bone phenotypes at P1 (Supplementary Tables S1 and S2). These data suggest that the Hand transcriptional factors exert specific functions in the NCCs and bone primordium to control the patterning of the jaws.

### *Hand2-* and *Hand1*-induced mandibular transformations are accompanied by oral tissue remodeling

Lesser *Hand2*^*NC*^ mutants than expected were obtained (Supplementary Table S1). To determine whether this was a consequence of decreased viability, we examined *Hand2*^*NC*^ embryos. Until embryonic day (E) 12.5, the surface appearance of *Hand2*^*NC*^ embryos was normal. At E13.5, *Hand2*^*NC*^ mutants were characterised externally by inadequate pigmentation in the ventral retina (Supplementary Fig. S3). At E13.5–E16.5, *Hand2*^*NC*^ mutants showed small pinna, brachycephaly, and tongue protrusion. In addition, *Hand2*^*NC*^ mutants often exhibited exencephaly or hemorrhage (Supplementary Fig. S3).

**Figure 4:**
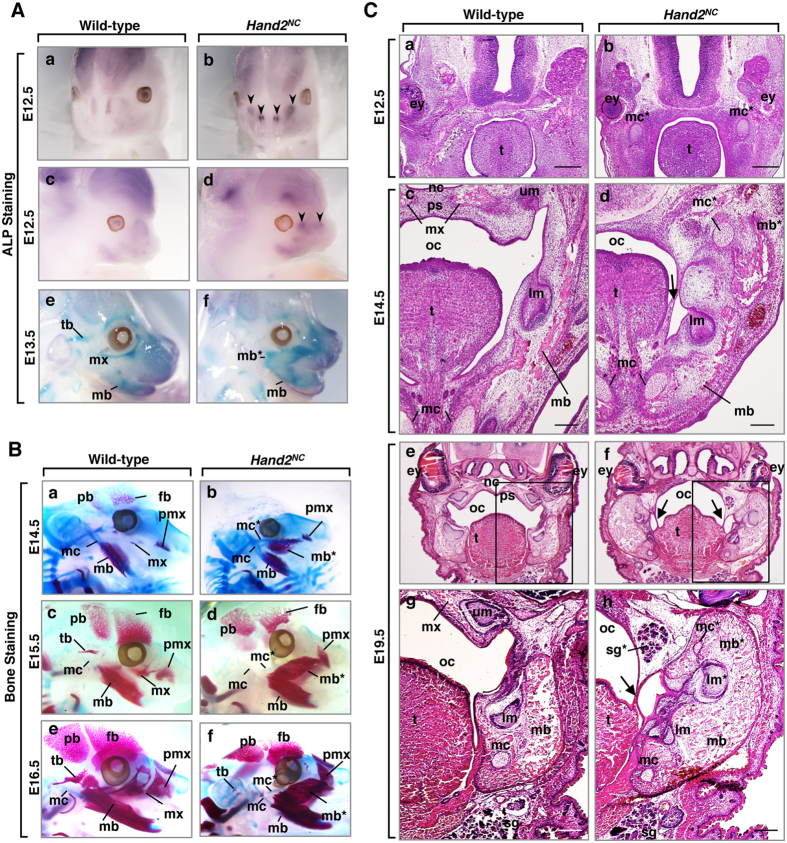
Morphological transformation of the maxillary to mandibular process in *Hand2*^*NC*^ mutants. (**A**) Whole-mount alkaline phosphatase (ALP) staining of E12.5 (a–d) and E13.5 (e,f) wild-type (a,c,e) and *Hand2*^*NC*^ (b,d,f) embryos. The arrowheads indicate the changes in ALP expression (b,d). (**B**) Skeletal preparations at E14.5 (a,b), E15.5 (c,d), and E16.5 (e,f) from wild-type (a,c,e) and *Hand2*^*NC*^ (b,d,f) embryos. The mutant maxilla (mx) is transformed to duplicated mandibular bone primordium (mb*) and is associated with the duplicated Meckel’s cartilage (mc*). The delayed ossification of the temporal bones (tb) and fontal bones (fb) are observed in *Hand2*^*NC*^ mutants (b,d,f). (***C***) Hematoxylin and eosin (H&E)-stained coronal sections of E12.5 (a,b), E14.5 (c,d), and E19.5 (e–h) wild-type (a,c,e,g) and *Hand2*^*NC*^ (b,d,f,h) heads. In *Hand2*^*NC*^ mutants, duplicated Meckel’s cartilages (mc*) are observed in the maxillary region (b,d,h). The palatal shelves (ps) are missing, and the original mandible is fused to the duplicated mandible by connective tissue strings (arrows in d,f,h). At E19.5, *Hand2*^*NC*^ mutants exhibit ectopic salivary glands (sg*) in the maxillary region and a second set of lower molars (lm*) in the duplicated mandible (mb*) (h). Scale bars: 300 μm (a,b), 200 μm (c,d,g,h). mx, maxilla; mb, mandibular bone; tb, temporal bone; mc, Meckel’s cartilage; fb, frontal bone; pb, parietal bone; pmx, premaxilla; ey, eye; oc, oral cavity; sg, salivary glands; lm, lower molar; um, upper molar; t, tongue. An asterisk indicates a duplicate.

To analyse the effects of Hand2 on the mandibular bone patterning, we next examined jaw development of wild-type and *Hand2*^*NC*^ embryos from E12.5 to E16.5. Altered expression of alkaline phosphatase (ALP), an early osteoblast marker, was observed in the maxillary process of *Hand2*^*NC*^ mutants (Fig. 4Ab,d), suggesting that the destination of the maxillary arch was determined as early as E12.5. The duplicated mandible in the maxillary region was confirmed in whole-mount ALP staining of *Hand2*^*NC*^ mutants at E13.5 (Fig. 4Af). Bone staining of embryos showed that the maxillary bone was aplastic, the premaxilla was deformed, and the duplicated mandible contained ectopic truncated Meckel’s cartilage in the maxillary region of *Hand2*^*NC*^ mutants (Fig. 4Bb,d,f). Ossification of the temporal bones was delayed in *Hand2*^*NC*^ mutants (Fig. 4Bb,d,f).

To further examine the role of Hand2 in jaw development, we performed histological analysis of *Hand2*^*NC*^ mutants. At E12.5, ectopic Meckel’s cartilage was observed in the maxillary region of *Hand2*^*NC*^ mutants (Fig. 4Cb). At E14.5, the control palatal shelves were elevated above the dorsum of the tongue and fused at the midline (Fig. 4Cc), whereas the palatal shelves of *Hand2*^*NC*^ mutants were missing, and the duplicated mandible was fused to the original mandible by connective tissue strings (Fig. 4Cd). At E19.5, heads of *Hand2*^*NC*^ mutants showed duplication of the mandibular bone and Meckel’s cartilage, ectopic salivary glands in the maxillary region, and a second set of lower molars in the duplicated mandible (Fig. 4Ch). *Hand1*^*NC*^ mutants (Supplementary Fig. S4) were histologically similar to *Hand2*^*NC*^ mutants (Fig. 4C). These results revealed that Hand proteins determine the mandibular identity and that correct Hand expression is required in the mandibular arch for normal palatogenesis to occur.

### Hand2 controls expression of *Pbx1* and *Runx1* in the first branchial arch

**Figure 5:**
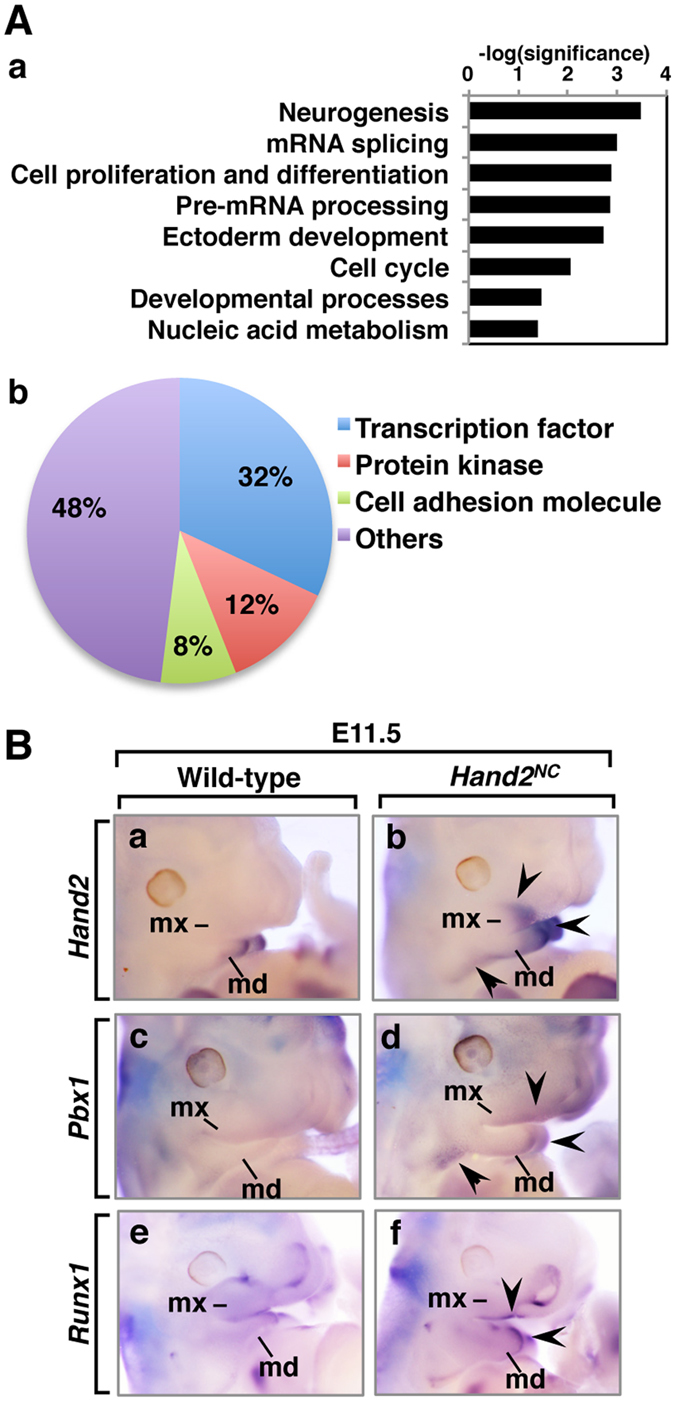
Aberrant gene expression resulting from the altered expression of *Hand2.* (**A**) Affinity GeneChip array analysis of wild-type and *Hand2*^*NC*^ heads at E11.5. Gene ontology analysis was performed with the PANTHER classification system (n = 4 per genotype). (a) Significantly (*P* < 0.05) enriched biological processes are shown. (b) The prominent molecular function in the developmental processes is the transcription factor (*P* = 0.00016). (**B**) The expression patterns of the genes that are upregulated in the *Hand2*^*NC*^ maxillary and mandibular processes at E11.5. Lateral views of wild-type (a,c,e) and *Hand2*^*NC*^ (b,d,f) embryos that were processed by whole-mount *in situ* hybridization. Arrowheads indicate changes in gene expression. md, mandibular arch; mx, maxillary arch.

The maxilla-to-mandible transformation of the first branchial arch in *Hand2*^*NC*^ mutants indicated that altered *Hand2* expression converted the genetic program from the maxillary to the mandibular arch. To further determine the downstream genes of Hand2, we performed gene expression profiling on E11.5 *Hand2*^*NC*^ embryos. Surprisingly, only a small portion of the genes was dysregulated in E11.5 *Hand2*^*NC*^mutants; 51 genes were downregulated, and 17 genes, including the *Hand2* gene, were upregulated when the two-fold change cutoff was applied (Supplementary Tables S3 and S4). To analyse whether certain molecular functions or biological processes were sensitive to the altered *Hand2* expression in the neural crest, a gene ontology enrichment analysis was performed on the dysregulated genes. Protein ANalysis THrough Evolutionary Relationships (PANTHER)^29^ indicated that the prominent biological process was neurogenesis (Fig. 5Aa). The genes that were dysregulated in the developmental processes were further analysed for their annotated molecular function. The majority of the dysregulated genes encoded transcription factors (Fig. 5Ab, Supplementary Table S5).

To understand the transcriptional network controlled by Hand2, we examined the dysregulated expression of the transcription factors in E11.5 embryos. In *Hand2*^*NC*^mutants, levels of *Hand2* and *Pbx1* expression were increased in the medial mandibular process and hyoid arch, and the altered expression of these genes was observed in the maxillary process (Fig. 5Bb,d). The *Runx1* expression was also increased in the medial mandibular process and maxillomandibular junction in *Hand2*^*NC*^mutants (Fig. 5Bf). Analysis of the expression of *Hif1a, Rsf1*, *Six4*, *Rb1cc1*, *Sfmbt2,* and *Dner* did not reveal any obvious differences between the wild-type and *Hand2*^*NC*^heads (Supplementary Fig. S5).

### Hand2 activates a mandible-specific genetic program in the maxillary process

**Figure 6:**
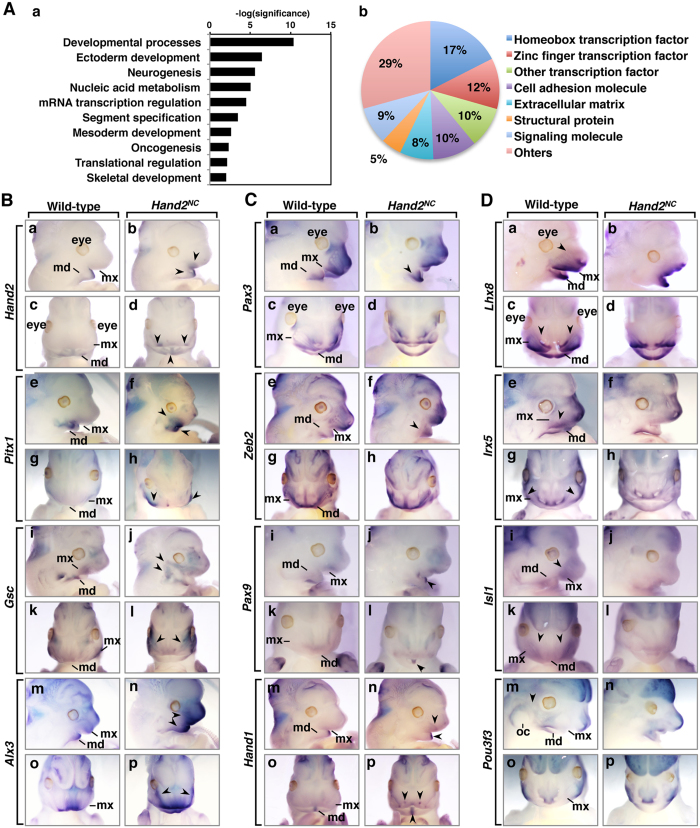
Hand2-regulated expression of homeobox transcription factors. (**A**) Affinity GeneChip array analysis of wild-type and *Hand2*^*NC*^ heads at E12.5 (n = 3 per genotype). Gene ontology analysis was performed with the PANTHER classification system. (a) Significantly (*P* < 0.05) enriched biological processes are shown. (b) The prominent molecular function in the developmental processes is the homeobox transcription factor (*P* = 8.1 × 10^−17^). (**B–D**) The expression patterns of the genes that are dysregulated in the *Hand2*^*NC*^ maxillary and mandibular processes at E12.5. Lateral (upper panels) and frontal (lower panels) views of wild-type and *Hand2*^*NC*^ embryos processed by whole-mount *in situ* hybridization, as indicated. Arrowheads indicate the changes in gene expression. md, mandibular process; mx, maxillary process; oc, otic capsule.

*Hand2* gene expression was confirmed in the mandibular region at E12.5 (Fig. 1B) and *Hand2*^*NC*^mutants were first recognised by dysmorphogenesis of the head at E12.5 (Fig. 4C). To find downstream genes of Hand2, we next performed a microarray analysis using the heads from E12.5 embryos. We found that 61 genes, including the *Hand2* gene, were upregulated, and 84 genes were downregulated, with a cutoff of a two-fold change (Supplementary Tables S6 and S7). Interestingly, the expression of *Smr2*, a member of the gene family encoding salivary glutamine/glutamic acid-rich proteins, was upregulated in *Hand2*^*NC*^ mutant heads at E12.5 (Supplementary Table S7). A gene ontology analysis showed that the most significantly enriched biological process in the E12.5 *Hand2*^*NC*^ mutants was the developmental processes (Fig. 6Aa). The prominent molecule was transcription factors, especially the homeobox transcription factor (Fig. 6Ab). Most dysregulated homeobox transcription factors have been linked to human genetic diseases or genetically engineered mice with craniofacial anomalies (Supplementary Table S8).

The patterning of cranial and branchial NCCs is achieved by the regulation of homeobox transcription factors. To better understand the transcriptional network that is controlled by Hand2 during jaw patterning, we focused on the homeobox transcription factors that had altered regulation in *Hand2*^*NC*^mutants. Whole-mount *in situ* hybridization showed that *Hand2* was only expressed in the mandibular process of wild-type embryos, whereas ectopic *Hand2* expression was observed in the lateral region of the maxillary process in *Hand2*^*NC*^ mutants at E12.5 (Fig. 6B). The ectopic expression of *Pitx1* was also confirmed in the maxillary process and increased in the mandibular process of *Hand2*^*NC*^ mutants (Fig. 6B). *Gsc* expression is downregulated in the branchial arches of *hand2* mutant zebrafish *han*^*S6*^ and conditional *Hand2* knockout mice^30^,^31^. In *Hand2*^*NC*^ mutants, *Gsc* was ectopically expressed, suggesting that Hand2 maintains or activates *Gsc* expression in the branchial arches. *Alx3* expression was increased in the mandibular and maxillary processes (Fig. 6B). The levels of expression of *Pax3* and *Zeb2* were increased in the mandibular process of *Hand2*^*NC*^ mutants (Fig. 6C). We have shown that the ablation of *Hand1* and *Hand2* in the NCCs affects the expression of *Pax9* in the distal midline of the mandibular process^10^. In *Hand2*^*NC*^ mutants, the levels of *Pax9* expression were increased in the midline of the mandibular process where *Hand2* expression was highly upregulated by the transgene (Fig. 6C). The expression of *Lhx8*, as well as *Isl1*, which is required for establishing the posterior hind limb field upstream of the *Hand2*-*Shh* pathway^32^, was less intense in the maxillary process of *Hand2*^*NC*^ mutants, while the expression of these genes was not affected in the mandibular process (Fig. 6D). Similarly, the expression of *Irx5*, which has been identified as a direct target of Hand2 in limb buds^24^, was reduced in the maxillary process (Fig. 6D). *Pou3f3* is required for the formation of maxillary components, including the temporal bone, jaw joint, and incus^33^. Consistent with the bone phenotype of *Hand2*^*NC*^mutants (Fig. 2A,C), *Pou3f3* expression was absent at the maxillary-mandibular junction in *Hand2*^*NC*^mutants (Fig. 6D). Because Hand2 regulates *Hand1* expression in the mandibular arch^31^, we examined *Hand1* expression in *Hand2*^*NC*^ mutants. Ectopic *Hand1* expression in the maxillary process of *Hand2*^*NC*^ mutants was confirmed in the lateral region, although the expression level appeared lower than that in the midline of the mandibular process (Fig. 6C). The expression of *Zfhx3*, *Hmx1*, and *Shox2* was not obviously different in the mandibular and maxillary processes of wild-type and *Hand2*^*NC*^ mutants (Supplementary Fig. S5).

**Figure 7:**
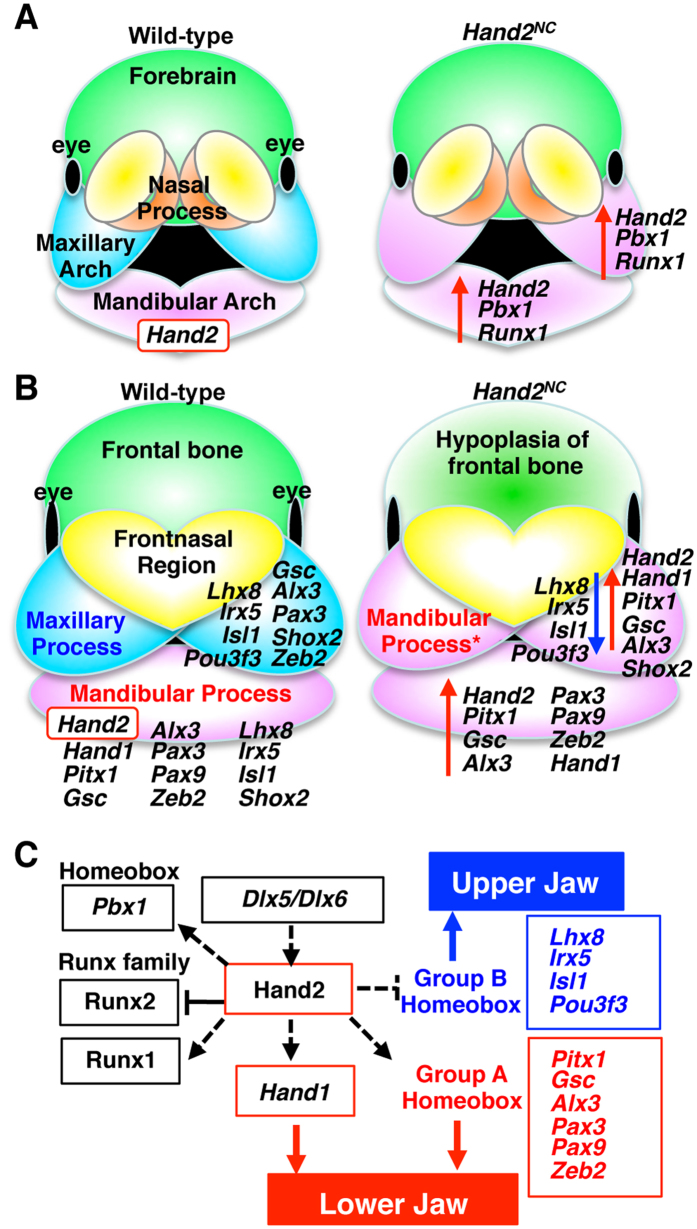
The putative networks governed by Hand2 in the branchial arch. (**A,B**) Schematic presentation of the transcription factor expression that is affected by altered *Hand2* expression in the neural crest-derived cells at E11.5 (A) and E12.5 (B). The red arrows indicate upregulation, and the blue arrow indicates downregulation in the *Hand2*^*NC*^ mutants compared with the wild-type. The nested expression of *Hand2* in the mandibular process regulates jaw patterning by controlling the homeobox transcription factors. An asterisk indicates a duplicate. (**C**) Model of the putative transcriptional regulation of mandibular identity by Hand2. Dlx5 and Dlx6 activate *Hand2* expression distally^15^,^16^,^33^. Hand2 induces and/or maintains Group A and represses Group B homeobox transcription factors in the mandibular process. Presumably, the Group A homeobox transcription factors are involved in lower jaw identity, whereas the Group B homeobox transcription factors promote upper jaw development. The solid lines indicate direct interactions, whereas the dashed lines indicate genetic interactions.

Oral functional registration of the palate requires the correct integration of the secondary palate of the maxillary process and the primary palate. For simultaneous breathing and feeding, palate formation between the oral and nasal cavities must be appropriately aligned. Cleft palate is the most frequent congenital anomaly in humans^34^. Altered *Hand2* gene expression in the maxillary arch results in a loss of the secondary palate and nasal cavities (Fig. 4C), suggesting that the dysregulated genes in *Hand2*^*NC*^ mutants are involved in palatogenesis. Whole-mount *in situ* hybridization of the E12.5 embryos also showed that *Alx3, Lhx8, Pax3, Pitx1*, *Shox2, Gsc, Zeb2, Isl1, Rhox4b, Uncx,* and *Hmx1* were expressed in the secondary palate (Supplementary Fig. S6), suggesting that these homeobox transcription factors may be implicated in palatal development as intrinsic factors. Among these genes, ablation or mutation of *Alx3, Lhx8, Pax3, Pitx1*, *Shox2,* and *Zeb2* induces cleft palates in humans and/or mice (Supplementary Table S8). Collectively, these results indicate that NCCs within the first branchial arches respond to Hand2 transcription activity, which controls jaw patterning and palatogenesis by regulating the expression of homeobox transcription factors (Fig. 7).

## Discussion

The bHLH transcription factor Hand2 is expressed in NCCs in the mandibular arch but not in the maxillary arch of the first branchial arch. The Hand2 sequence is conserved among jawed vertebrates but not among jawless vertebrates. Consistent with its expression pattern and amino-acid alignment, *Hand2* gene expression is incompatible with maxillary jaw development, and altered Hand2 expression transforms the maxilla to the mandible and controls the specification of jaw identity. Hand2 orchestrates the dynamics of transcription factors and regulates the patterning of the first branchial arch (Fig. 7), suggesting that its role as one of the central molecules that determine the dorsoventral polarity of the first branchial arch. The skeletal abnormalities of *Hand2*^*NC*^mutants were similar to those seen in *Ednra*^*Hand2/*+^ mutants^19^ except for the penetration. However, we cannot formally rule out the possibility that overexpression of *Hand2* may lead to changes that are not physiological and that are not representative of the true function or network of Hand2.

### Regulation of jaw patterning and bone formation by Hand2

The functional modification of genes has been causally linked to adaptive evolution. The Hand2 amino sequence is evolutionally conserved in jawed vertebrates but less conserved in jawless vertebrates and invertebrates. In particular, the N-terminus of Hand2 is only conserved among jawed vertebrates, suggesting that the domain may contribute to the acquisition of the hinged jaw and the evolution from jawless to jawed vertebrates. We have shown that the N-terminus of Hand2 has an anti-osteogenic function by directly inhibiting Runx2^11^. Interestingly, *hand2* zebrafish mutant *han*^*c99*^, which lacks a region equivalent to the N-terminal amino acids (1–53aa) of mouse Hand2, has defects in jaw development and exhibits continuous upper jaw joints^35^,^36^. Since Hand proteins can dimerise with bHLH proteins to form a bipartite DNA-binding domain that recognises the E-box element^23^,^37^,^38^, the overexpression of Hand proteins may alter the dimerization pool of bHLH transcription factors or the DNA-binding activity in the branchial arch. The expression patterns of *hand* in the branchial arches of lampreys are similar to those of orthologous *hand* genes in jawed vertebrates^22^, even though the number of *Hand* orthologs expressed in the branchial arches of jawless and jawed vertebrates may be different. Multiple-species sequence alignment and expression analysis of Hand2 target molecules may yield insights into vertebrate evolution.

Transformation of the upper jaw into the lower jaw is observed in *Edn1* knock-in mice (*Ednra*^*Edn1/*+^), in which one copy of *Edn1* was knocked into the *Ednra* locus, resulting in constitutive *Ednra* activation^19^. The effects of constitutive *Ednra* activation are mostly restricted to the first branchial arch, even though *Ednra* expression occurs throughout the head and branchial arches^19^. In *Ednra*^*Edn1/*+^ mice, the complete mandibular bones with continuous Meckel’s cartilage and malleus were duplicated. In *Hand2*^*NC*^ mutants, the duplicated dentary was complete with the angular process, but the condylar process, coronoid process, and malleus were aplastic or hypomorphic in the duplicated mandible, suggesting that *Hand2* is involved in the formation of the dentary and angular process of the mandibular bone. In the maxillary region of *Hand2*^*NC*^ mutants, the presphenoid, vomer, and palatal bones were missing, and they were replaced by mandibular elements, whereas these maxillary elements remained in *Ednra*^*Edn1/*+^ mice. It is possible that the constitutive *Ednra* activation predominantly affects the first branchial arch in *Ednra*^*Edn1/*+^ mice, whereas the expression of *Hand2* is altered in the cranial NCCs in *Hand2*^*NC*^mice. Other explanations are that part of the *Hand2* expression is independent from *Ednra1/Edn1*-*Dlx5/6* signalling^17^,^19^.

The altered expression of *Hand2* in the cranial NCC-derived bone primordium resulted in hypoplastic bone formation in the cranial region, and this was observed in the frontal bones, temporal bones, and dorsal mandibular elements. Altered *Hand2* expression in the bone primordium (*Hand2*^*BP*^) resulted in the aplasia of the basioccipital bones. These results indicate that Hand2 antagonises the genetic program for the formation of membranous bones and are consistent with the findings that Hand2 negatively regulates the ossification of the mandibular bone by directly inhibiting Runx2, which is a master transcription factor of osteoblast-specific genes^11^. In contrast, the response of the NCCs in the second branchial arch to the altered *Hand2* expression was limited, suggesting that the levels of *Hand2* expression were not critical in the second branchial arch. Because the targeted deletion of *Hoxa2* transforms the second branchial arch to a mandibular branchial arch^39^, *Hox* genes may be primarily involved in determining the patterning of the second branchial arch. Thus, the *Hand2* expression levels and domains must be exquisitely regulated for the bone formation of the first branchial arch.

### Regulation of gene expression by Hand2

The present studies demonstrate that a number of genes are disrupted following the *Hand2* overexpression, subsequently inducing the maxilla-to-mandible transformation. The transformation induced a mirror-image duplication, suggesting that positional information along the maxillary-mandibular axis is present. *Runx1*, which is expressed in the maxillomandibular junction of the first branchial arch, may function as one of the patterning molecules. Interestingly, some of the dysregulated genes in the E11.5 *Hand2*^*NC*^ mice are involved in the transformation of elements. In *Pbx1*-deficient mice, the NCC-derived bones of the second branchial arch were morphologically transformed into elements that were reminiscent of first arch-derived cartilage^40^. In *Hoxc8*-deficient mice, several skeletal segments were transformed into the likeness of more anterior ones^41^. At E12.5, Hand2-mediated signalling positively regulated the mandibular-specific expression of *Pitx1, Gsc, Alx3, Zeb2*, and *Pax9* and influenced the repression of *Lhx8, Irx5, Isl1,* and *Pou3f3* genes in the mandibular arch. Osterwalder *et al.* (2014) identified a set of Hand2-target regions in mouse embryonic tissues with Hand2 chromatin immunoprecipitation (ChIP)-qPCR analysis^24^, and showed that Hand2 chromatin complexes were enriched in the genomic landscape of the following transcription regulators: *Pbx1, Pitx1, Gsc, Alx3, Shox2, Pax3, Zeb2, Pax9, Lhx8, Irx5, Isl1, Pou3f3,* and *Hand1* (Gene Expression Omnibus #GSE55707). Hand2 may directly regulate some of the transcription factors that participate in craniofacial development. The premaxilla of *Hand2*^*NC*^ mutants remained with upper incisors, and the ectopic induction of transcription factors was not observed in the midline of the maxillary process, suggesting that the effects of *Hand2* were restricted to the maxillary prominences, except in the FNP. In *Hand2*^*NC*^ mutants, the environmental signals from the FNP ectodermal zone may competently respond to Hand2 signalling.

### Implications for human diseases

The homeobox transcription factors with altered regulation in *Hand2*^*NC*^ mutants are also important in the etiology of the Mendelian forms of syndromes. *Alx3, Gsc, Pax3, Pax9, Pitx1*, and *Zeb2*, the expression of which was positively regulated by Hand2-mediated signalling, are the disease genes of craniofacial syndromes (Supplementary Table S8). Among the craniofacial syndromes, auriculocondylar syndrome [Online Mendelian Inheritance in Man (OMIM) #602483 and #614669] is characterised by outer ear and mandible malformations that affect NCC development within the first and second branchial arches. Recent studies have found that patients with auriculocondylar syndrome have mutations in the genes, *EDN1*, *EDNRA1*, *PLCB4* and *GNAI3*, which function in the endothelin signalling pathway^42^,^43^,^44^; however, mutations in *HAND2* have not been identified in patients with auriculocondylar syndrome. Since the homeobox transcription factors with altered regulation in *Hand2*^*NC*^ mutants were also expressed in the palatal shelves, the mutations in genes downstream of Hand2 could also be associated with craniofacial syndromes with mandibular and/or palatal anomalies.

In summary, our analysis reveals that altered *Hand2* expression in the maxillary arch results in altered homeobox transcription factor regulation in the mandibular and maxillary processes and a transformation of the upper jaw into the lower jaw. The results of the present study also suggest that nested *Hand2* expression in the mandibular arch is necessary for palatogenesis. *Hand2* and *Hand1* genes might have had a central role in the coordination of oral morphogenesis that diverted the craniofacial shape noted in vertebrates and the transition between filter feeding and active predation by providing specific mandibular identity. Moreover, the modification of the Hand2 sequence may contain a specific code that contributed to the transition from jawless to jawed vertebrates. Integration of the molecular data will enhance the understanding of the morphogenetic program for mandibular and maxillary patterning.

## Methods

### Generation of *Hand2* and *Hand1*-mutant mice

The transgene vectors *CAG-lox-CAT-lox-Hand2-polyA* and *CAG-lox-CAT-lox-Hand1-polyA* were constructed and injected into fertilised eggs to generate the permanent transgenic lines *CAG-CAT Hand2*^*Tg/*+^ (Stock No. RBRC01366, RIKEN, Wako, Saitama, Japan) and *CAG-CAT Hand1*^*Tg/*+^ (Stock No. RBRC01369, RIKEN), respectively. *Hand2-LacZ*^45^, *Wnt1-Cre*^46^ (Stock No. 7807, The Jackson Laboratory, Bar Harbor, ME, USA), *Twist2-Cre* knock-in^26^,^47^ (Stock No. 8712, The Jackson Laboratory), and *KRT14-Cre*^48^ (Stock No. 4782, The Jackson Laboratory) mice have been previously described. For the conditional activation of *Hand2* or *Hand1*, we generated double-transgenic mice by intercrossing *CAG-floxed CAT-Hand2* or *-Hand1* with *Wnt1-Cre, Twist2-Cre*, or *KRT14-Cre* mice. Wild-type littermates were used as controls. All of the animal experimental procedures were reviewed and approved by the Institutional Animal Care and Use Committees of the Tokyo Medical and Dental University and the University of Texas Southwestern Medical Center (Permit Number: 0160215A). All experiments were carried out in accordance with the approved guidelines.

### Histology and *in situ* hybridization

The embryos were fixed in 4% paraformaldehyde/phosphate buffered saline and embedded in paraffin. The sections were stained with hematoxylin & eosin (H&E). Whole-mount and section *in situ* hybridization was performed as described previously^11^,^28^. A probe for *Hand1* was generously provided by Eric N. Olson^49^. Other probes were prepared by reverse transcription (RT)-PCR. RT-PCR was performed as described previously^50^. The primers used for RT-PCR are listed in Supplementary Table S9.

### Ortholog identification

The Hand2 protein sequences of the following 10 vertebrates and 2 invertebrates were downloaded from the Ensembl Genome Browser (http://asia.ensembl.org/index.html): human, mouse, chicken, zebra finch, *Xenopus tropicalis*, medaka, stickleback, *Tetraodon*, cod, zebrafish, fruit fly, and *Caenorhabditis elegans*. The elephant shark proteome was obtained from the Elephant Shark Genome Project (http://esharkgenome.imcb.a-star.edu.sg)^5^. To identify the ortholog of the lamprey, a protein Basic Local Alignment Search Tool (BLAST) was run with default settings from the Japanese Lamprey Genome Project (http://jlampreygenome.imcb.a-star.edu.sg). The BLAST analysis of the amino-acid sequences of the mouse Hand proteins against the entire lamprey genome revealed no appreciable identity to any of the sequences except for *JL1799*. ClustalW (http://www.genome.jp/tools/clustalw) and Ensembl were used to create multiple sequence alignments of Hand2 and the rooted phylogenetic trees of the Hand proteins.

### Skeletal staining, β-galactosidase staining, and whole-mount ALP staining

Alizarin red and alcian blue staining, β-galactosidase staining, and whole-mount ALP staining were performed as described previously^11^,^28^. The representative pictures from each group (n = 3) were shown.

### Microarray and gene ontology analysis

Total RNA was extracted either from wild-type or *Hand2*^*NC*^ embryos with TRIzol (Thermo Fisher Scientific Inc., Waltham, MA, USA) and a RNeasy Mini Kit (QIAGEN GmbH, Hilden, Germany) and subsequently pooled prior to analysis. For each RNA sample, the concentration and purity were measured with a NanoDrop ND-1000 spectrometer, and the quality was then checked with 2100 Bioanalyzer (Agilent Technologies, Inc., Santa Clara, CA, USA). The microarray analysis was performed by the KURABO Bio-Medical Department with the GeneChip Mouse Genome 430 2.0 Array (Affymetrix, Inc., Santa Clara, CA, USA). A gene ontology analysis was performed with the PANTHER database (http://pantherdb.org). Only biological processes with significant (*P* < 0.05) enrichment were used to examine molecular function. To elucidate the molecular pathogenesis of genes, genetic diseases in humans and genetically engineered mice were investigated with the OMIM program (http://omim.org) and Mouse Genome Informatics program (http://www.informatics.jax.org), respectively.

## Additional Information

**How to cite this article**: Funato, N. *et al.* Specification of jaw identity by the Hand2 transcription factor. *Sci. Rep.* **6**, 28405; doi: 10.1038/srep28405 (2016).

## Supplementary Material

Supplementary Information
