# Supplementary material for: Specification of jaw identity by the Hand2 transcription factor
Source: Sci Rep. 2016 Jun 22;6:28405. doi: 10.1038/srep28405 (PMC4916603; doi:10.1038/srep28405)
Supplement: Supplementary Information [file srep28405-s1.pdf]

Specification of jaw identity by the Hand2 transcription factor

Noriko Funato, Hiroki Kokubo, Masataka Nakamura, Hiromi Yanagisawa, and Yumiko Saga

Supplementary Information

Supplementary Table S1. Analysis of the tissue-specific induction of *Hand2* and *Hand1* with the indicated *Cre* driver.

	Neural Crest		Bone primordium		Epithelium
	Hand2$^{CAT/+}$; *Wnt1-Cre* (*Hand2*^{NC})	*Hand1*$^{CAT/+}$; *Wnt1-Cre* (*Hand1*^{NC})	*Hand2*$^{CAT/+}$; *Twist2-Cre* (*Hand2*^{BP})	*Hand1*$^{CAT/+}$; *Twist2-Cre* (*Hand1*^{BP})	*Hand2*$^{CAT/+}$; *KRT14-Cre*
**Neonatal lethality**	+	+	+	–	–
	100% (7/7)	100% (5/5)	100% (7/7)	(n=13)	(n=5)
**Surface appearance at P1**					
**Brachycephaly**	+	+	+	+	–
	100% (7/7)	100% (5/5)	100% (7/7)	100% (13/13)	(n=5)
**Hypoplastic ears**	+	+	+	–	–
	100% (7/7)	100% (5/5)	100% (7/7)	(n=13)	(n=5)
**Open eye lids**	+	+	+	+	–
	100% (7/7)	100% (5/5)	100% (7/7)	100% (13/13)	(n=5)
**Mutant frequency at P1**	7.7%	12.8%	21.9%	20.6%	20.8%
	(11/142)	(5/39)	(7/32)	(13/63)	(5/24)
**Expected frequency**	25%	25%	25%	25%	25%

–, no apparent phenotype.

Supplementary Table S2. The bone phenotypes of the tissue-specific induction of *Hand1* or *Hand2* with the indicated *Cre* driver.

		Neural Crest Cells		Bone primordium		Epithelium
		Hand2$^{CAT/+}$; Wnt1-Cre (*Hand2^{NC}*)	*Hand1$^{CAT/+}$; Wnt1-Cre* (*Hand1^{NC}*)	*Hand2$^{CAT/+}$; Twist2-Cre* (*Hand2^{BP}*)	*Hand1$^{CAT/+}$; Twist2-Cre* (*Hand1^{BP}*)	*Hand2$^{CAT/+}$; KRT14-Cre*
**Mandibular process**	**Mandibular bone**	duplication 100% (5/5)	partial duplication 100% (n=3)	partial duplication 100% (n=7)	– (n=3)	– (n=5)
	**mandibular dentary**	hypoplasia 100% (5/5)	hypoplasia 100% (3/3)	hypoplasia 100% (7/7)	hypoplasia 100% (3/3)	– (n=5)
	**lower incisor**	duplication 100% (5/5)	– (n=3)	– (n=7)	– (n=3)	– (n=5)
	**angular process**	hypoplasia 100% (5/5)	– (n=3)	hypoplasia 100% (7/7)	– (n=3)	– (n=5)
	**coronoid process**	aplasia 100% (5/5)	– (n=3)	hypoplasia 100% (7/7)	– (n=3)	– (n=5)
	**condylar process**	aplasia 100% (5/5)	– (n=3)	hypoplasia 100% (7/7)	– (n=3)	– (n=5)
	**Tympanic ring**	aplasia 100% (5/5)	hypoplasia 100% (3/3)	aplasia/ hypoplasia 100% (7/7)	hypoplasia 100% (3/3)	– (n=5)
	**Gonial bone**	aplasia 100% (5/5)	– (n=3)	aplasia 100% (7/7)	– (n=3)	– (n=5)
**Maxillary process**	**Maxillary bone**					
	**palatal process of maxilla**	aplasia 100% (5/5)	aplasia 100% (3/3)	aplasia 100% (7/7)	– (n=3)	– (n=5)
	**palatal process of palatine**	aplasia 100% (5/5)	aplasia 100% (3/3)	aplasia 100% (7/7)	– (n=3)	– (n=5)
	**frontal process of maxilla**	hypoplasia 100%(5/5)	hypoplasia 100% (3/3)	hypoplasia 100% (7/7)	– (n=3)	– (n=5)
	**Jugal**	aplasia 100% (5/5)	hypoplasia 100% (3/3)	aplasia 100% (7/7)	– (n=3)	– (n=5)
	**Lamina obturans**	aplasia 100% (5/5)	hypoplasia 100% (3/3)	hypoplasia 100% (7/7)	– (n=3)	– (n=5)
	**Pterygoid bone**	aplasia 100% (5/5)	aplasia 100% (3/3)	hypoplasia 100% (7/7)	hypoplasia 100% (3/3)	– (n=5)
	**Temporal bone**	hypoplasia 100% (5/5)	hypoplasia 100% (3/3)	hypoplasia 100% (7/7)	– (n=3)	– (n=5)
**Frontonasal**	**Premaxilla**	deformed	deformed	–	–	–

**process**		(n=5)	(n=3)	(n=7)	(n=3)	(n=5)
	**upper incisor**	–	–	–	–	–
		(n=5)	(n=3)	(n=7)	(n=3)	(n=5)
**Neurocranium**	**Presphenoid bone**	aplasia	aplasia	hypoplasia	hypoplasia	–
		100% (5/5)	100% (3/3)	100% (7/7)	100% (3/3)	(n=5)
	**Basisphenoid bone**	hypoplasia	hypoplasia	hypoplasia	hypoplasia	–
		100% (5/5)	100% (3/3)	100% (7/7)	100% (3/3)	(n=5)
	**Basioccipital bone**	–	–	aplasia	hypoplasia	–
		(n=5)	(n=3)	100% (7/7)	100% (3/3)	(n=5)
**Calvaria**	**Frontal bone**	hypoplasia	hypoplasia	hypoplasia	–	–
		100% (5/5)	100% (3/3)	100% (7/7)	(n=3)	(n=5)
	**Parietal bone**	–	–	–	–	–
		(n=5)	(n=3)	(n=7)	(n=3)	(n=5)
**Second**	**Hyoid bone**	–	hypoplasia	–	–	–
**branchial arch**		(n=5)	33% (1/3)	(n=7)	(n=3)	(n=5)

–, no apparent phenotype by bone staining.

Supplementary Table S3. Genes with decreased expression in the E11.5 *Hand2*^{NC} samples.

Fold Change	Gene Symbol	Gene Title	Entrez Gene ID
0.0	***Mudeng***	MU-2/AP1M2 domain containing, death-inducing	74385
0.0	***Slc5a5***	solute carrier family 5 (sodium iodide symporter), member 5	114479
0.0	***Cetn1***	centrin 1	26369
0.0	***C87414***	expressed sequence C87414	381654
0.0	***4930532I03Rik***	RIKEN cDNA 4930532I03 gene	75833
0.1	***B230209E15Rik***	RIKEN cDNA B230209E15 gene	319752
0.1	***Slxl1***	Slx-like 1	75140
0.1	***Map4k3***	mitogen-activated protein kinase kinase kinase kinase 3	225028
0.1	***Slc22a29***	solute carrier family 22. member 29	236293
0.1	***Slco1b2***	solute carrier organic anion transporter family, member 1b2	28253
0.1	***Tnfsf13b***	tumor necrosis factor (ligand) superfamily, member 13b	24099
0.1	***Krtap5-2***	keratin associated protein 5-2	71623
0.1	***S100a3***	S100 calcium binding protein A3	20197
0.1	***Calm4***	calmodulin 4	80796
0.1	***Mif***	macrophage migration inhibitory factor	17319
0.1	***Hrh1***	histamine receptor H1	15465
0.1	***Prss37***	protease, serine, 37	67690
0.1	***Abca14***	ATP-binding cassette, sub-family A (ABC1), member 14	67928
0.1	***1700066J24Rik***	RIKEN cDNA 1700066J24 gene	76992
0.1	***Gm9999***	predicted gene 9999	629141
0.1	***Paqr5***	progestin and adipoQ receptor family member V	74090
0.1	***Ubqln2***	ubiquilin 2	54609
0.1	***9030405F24Rik***	RIKEN cDNA 9030405F24 gene	74531
0.1	***C1ra***	complement component 1, r subcomponent A	50909
0.2	***Tmsb15a***	thymosin beta 15a	78478
0.2	***Pcdh12***	protocadherin 12	53601
0.2	***Pisd-ps3***	phosphatidylserine decarboxylase, pseudogene 3	66776
0.2	***Sva***	seminal vesicle antigen	20939
0.2	***Klkb1***	kallikrein B, plasma 1	16621
0.2	***Adam2***	a disintegrin and metallopeptidase domain 2	11495
0.2	***Mog***	myelin oligodendrocyte glycoprotein	17441
0.2	***Car8***	carbonic anhydrase 8	12319
0.2	***Pank1***	pantothenate kinase 1	75735
0.2	***Tdo2***	tryptophan 2,3-dioxygenase	56720
0.2	***Impact***	imprinted and ancient	16210
0.2	***Ppt1***	palmitoyl-protein thioesterase 1	19063
0.3	***Amdhd1***	amidohydrolase domain containing 1	71761
0.3	***Ear4***	eosinophil-associated, ribonuclease A family, member 4	53877

0.3	***Ncoa2***	nuclear receptor coactivator 2	17978
0.3	***Arhgap4***	Rho GTPase activating protein 4	171207
0.3	***Setd6***	SET domain containing 6	66083
0.3	***Rnaset2a***	ribonuclease T2A	100037283
0.4	***4930429F24Rik***	RIKEN cDNA 4930429F24 gene	74633
0.4	***Tmed6***	transmembrane emp24 protein transport domain containing 6	66269
0.4	***Stk33***	serine/threonine kinase 33	117229
0.4	***2810417H13Rik***	RIKEN cDNA 2810417H13 gene	68026
0.4	***4930445B03Rik***	RIKEN cDNA 4930445B03 gene	74867
0.4	***Snn***	stannin	20621
0.5	***Mc1r***	melanocortin 1 receptor	17199
0.5	***Arf1***	ADP-ribosylation factor 1	11840
0.5	***Map3k6***	mitogen-activated protein kinase kinase kinase 6	53608

Supplementary Table S4. Genes with increased expression in the E11.5 *Hand2*^{NC} samples.

Fold Change	Gene Symbol	Gene Title	Entrez Gene ID
19.7	***Lat2***	linker for activation of T cells family, member 2	56743
18.4	***Icos***	inducible T cell co-stimulator	54167
18.4	***Pot1a***	protection of telomeres 1A	101185
14.9	***1700015G11Rik***	RIKEN cDNA 1700015G11 gene	100503036
14.9	***Ccdc7***	coiled-coil domain containing 7	74703
12.1	***Prl7a1***	prolactin family 7, subfamily a, member 1	19113
11.3	***Polr3a***	polymerase (RNA) III (DNA directed) polypeptide A	218832
8.6	***Apol9a***	apolipoprotein L 9a	223672
5.7	***Casp9***	caspase 9	12371
4.0	***Pcgf5***	polycomb group ring finger 5	76073
3.7	***Hand2***	heart and neural crest derivatives expressed transcript 2	15111
3.5	***Mrpl47***	mitochondrial ribosomal protein L47	74600
3.2	***Tlr12***	toll-like receptor 12	384059
3.0	***Slc18a1***	solute carrier family 18 (vesicular monoamine), member 1	110877
2.8	***9130002K18Rik***	RIKEN cDNA 9130002K18 gene	74554
2.5	***Fbrsl1***	fibrosin-like 1	381668
2.5	***Stx8***	syntaxin 8	55943
2.5	***Trp53bp1***	transformation related protein 53 binding protein 1	27223
2.1	***Dip2b***	DIP2 disco-interacting protein 2 homolog B (Drosophila)	239667
2.0	***BB163080***	expressed sequence BB163080	106459

Supplementary Table S5. Dysregulated transcription factors in E11.5 *Hand2^{NC}* embryos.

Gene	Protein	OMIM	Human genetic disease	Mutant mice with craniofacial phenotypes
Atf4	activating transcription factor 4	*604064	n.r.	Abnormal neurocranium morphology [1]
Dner	delta- and notch-like epidermal growth factor-related receptor	*607299	n.r.	n.r.
Fli1	Friend leukemia virus integration 1	*193067	n.r.	n.r.
Hand2	heart- and neural crest derivatives-expressed 2	*602407	n.r.	Small mandible, CP[2]
Hif1a	hypoxia-inducible factor 1, alpha subunit	*603348	n.r.	Absent pharyngeal arch arteries[3]
Hoxa5	homeobox A5	*142952	n.r.	Delayed ear emergence[4]
Hoxb3	homeobox B3	*142966	n.r.	n.r.
Hoxc8	homeobox C8	*142970	n.r.	n.r.
Hoxd1	homeobox D1	*142987	Clubfoot<br>Syndactyly	Fusion of atlas and occipital bones[5]
Meis2	Meis1, mouse, homolog of, 2	*601740	n.r.	n.r.
Pbx1	pre-B-cell leukemia transcription factor 1	*176310	Leukemia, acute pre-B-cell	Abnormal craniofacial bone morphology,<br>Palatal shelf hypoplasia,<br>Short snout[6,7]
Phox2b	paired-like homeobox 2b	*603851	Central hypoventilation syndrome	n.r.
Rb1cc1	RB1-inducible coiled-coil 1	*606837	n.r.	n.r.
Rsf1	repressor splicing factor 1	n.r.	n.r.	n.r.
Runx1	runt related transcription factor 1	*151385	Leukemia, acute myeloid<br>platelet disorder, familial, with associated myeloid malignancy	n.r.
Sfmbt2	Scm-like protein with 4 MBT domains 2	*615392	n.r.	n.r.
Six4	sine oculis-related homeobox 4	*606342	Posterior polar cataract<br>Myocardial stunning	No phenotype[8]

OMIM, Online Mendelian Inheritance in Man (http://omim.org); n.r., not reported; CP, cleft palate.
The homeobox transcription factors are in green.

Supplementary Table S6. Genes with decreased expression in E12.5 *Hand2*^{NC} samples.

Fold Change	Gene Symbol	Gene Title	Entrez Gene ID
0.0	***Ddx3y***	DEAD (Asp-Glu-Ala-Asp) box polypeptide 3, Y-linked	26900
0.0	***Uty***	ubiquitously transcribed tetratricopeptide repeat gene, Y chromosome	22290
0.0	***Eif2s3y***	eukaryotic translation initiation factor 2, subunit 3, Y-linked	26908
0.0	***Chmp4c***	charged multivesicular body protein 4C	66371
0.0	***Kdm5d***	lysine (K)-specific demethylase 5D	20592
0.0	***4933411E06Rik***	RIKEN cDNA 4933411E06 gene	71181
0.0	***Dhx9***	DEAH (Asp-Glu-Ala-His) box polypeptide 9	13211
0.1	***A230048O21Rik***	RIKEN cDNA A230048O21 gene	320959
0.1	***AY702102***	cDNA sequence AY702102	446211
0.1	***4930423D22Rik***	RIKEN cDNA 4930423D22 gene	74634
0.1	***Vmn1r25***	vomeronasal 1 receptor 25	113865
0.1	***Mpdz***	multiple PDZ domain protein	17475
0.1	***Lingo2***	leucine rich repeat and Ig domain containing 2	242384
0.1	***4930488N15Rik***	RIKEN cDNA 4930488N15 gene	75032
0.1	***Pou3f3***	POU domain, class 3, transcription factor 3	18993
0.1	***Aff4***	AF4/FMR2 family, member 4	93736
0.1	***Lipm***	lipase, family member M	78753
0.1	***Zfp369***	zinc finger protein 369	170936
0.1	***2900042E19Rik***	RIKEN cDNA 2900042E19 gene	72941
0.1	***Tmem150b***	transmembrane protein 150B	330460
0.1	***Cd200r1***	CD200 receptor 1	57781
0.1	***A930012N16Rik***	RIKEN cDNA A930012N16 gene	77813
0.1	***Bnip2***	BCL2/adenovirus E1B interacting protein 2	12175
0.1	***6330575P09Rik***	RIKEN cDNA 6330575P09 gene	76196
0.1	***Lrat***	lecithin-retinol acyltransferase	79235
0.1	***Tet1***	tet methylcytosine dioxygenase 1	52463
0.2	***Adrbk2***	adrenergic receptor kinase, beta 2	320129
0.2	***Timm9***	translocase of inner mitochondrial membrane 9 homolog (yeast)	30056
0.2	***1700126A01Rik***	RIKEN cDNA 1700126A01 gene	73602
0.2	***Tnn***	tenascin N	329278
0.2	***Adam33***	a disintegrin and metallopeptidase domain 33	110751
0.2	***Slc18a1***	solute carrier family 18 (vesicular monoamine), member 1	110877
0.2	***Snx6***	sorting nexin 6	72183
0.3	***4931431B13Rik***	RIKEN cDNA 4931431B13 gene	70973
0.3	***D12Ertd208e***	DNA segment, Chr 12, ERATO Doi 208, expressed	52495
0.3	***Rit2***	Ras-like without CAAX 2	19762
0.3	***Helz***	helicase with zinc finger domain	78455
0.3	***Naif1***	nuclear apoptosis inducing factor 1	71254
0.3	***Kdm4a***	lysine (K)-specific demethylase 4A	230674
0.3	***4831440E17Rik***	RIKEN cDNA 4831440E17 gene	320965

0.3	***A730009E18Rik***	RIKEN cDNA A730009E18 gene	319603
0.3	***Aqr***	aquarius	11834
0.3	***Peli1***	pellino 1	67245
0.4	***Alpi***	alkaline phosphatase, intestinal	76768
0.4	***3110040M04Rik***	RIKEN cDNA 3110040M04 gene	73176
0.4	***Msr1***	macrophage scavenger receptor 1	20288
0.4	***Pdx1***	pancreatic and duodenal homeobox 1	18609
0.4	***Rec8***	REC8 homolog (yeast)	56739
0.4	***Eif4g3***	Eukaryotic translation initiation factor 4 gamma, 3	230861
0.4	***Kif20b***	kinesin family member 20B	240641
0.4	***Prdm16***	PR domain containing 16	70673
0.4	***Rab3c***	RAB3C, member RAS oncogene family	67295
0.4	***Slc25a29***	solute carrier family 25, member 29	214663
0.4	***Stat1***	signal transducer and activator of transcription 1	20846
0.4	***Abi3bp***	ABI gene family, member 3 (NESH) binding protein	320712
0.4	***D830026I12Rik***	RIKEN cDNA D830026I12 gene	319682
0.4	***Gmfb***	glia maturation factor, beta	63985
0.4	***Mal***	myelin and lymphocyte protein, T cell differentiation protein	17153
0.4	***Mga***	MAX gene associated	29808
0.4	***Vamp5***	vesicle-associated membrane protein 5	53620
0.4	***Wdfy1***	WD repeat and FYVE domain containing 1	69368
0.5	***1700001P01Rik***	RIKEN cDNA 1700001P01 gene	72215
0.5	***4922501L14Rik***	RIKEN cDNA 4922501L14 gene	209601
0.5	***6430711C07Rik***	RIKEN cDNA 6430711C07 gene	76225
0.5	***A230055J12Rik***	RIKEN cDNA A230055J12 gene	320314
0.5	***Bach1***	BTB and CNC homology 1	12013
0.5	***Hdlbp***	high density lipoprotein (HDL) binding protein	110611
0.5	***Kank2***	KN motif and ankyrin repeat domains 2	235041
0.5	***Lsm11***	U7 snRNP-specific Sm-like protein LSM11	72290
0.5	***Nipbl***	Nipped-B homolog (Drosophila)	71175
0.5	***Nrcam***	neuron-glia-CAM-related cell adhesion molecule	319504
0.5	***Ociad1***	OCIA domain containing 1	68095
0.5	***Rhobtb3***	Rho-related BTB domain containing 3	73296
0.5	***Zeb2***	zinc finger E-box binding homeobox 2	24136
0.5	***2010106G01Rik***	RIKEN cDNA 2010106G01 gene	66552
0.5	***6720422M22Rik***	RIKEN cDNA 6720422M22 gene	99946
0.5	***Arpp21***	cyclic AMP-regulated phosphoprotein, 21	74100
0.5	***Atp6v0a1***	ATPase, H+ transporting, lysosomal V0 subunit A1	11975
0.5	***E2f6***	E2F transcription factor 6	50496
0.5	***Mef2c***	myocyte enhancer factor 2C	17260
0.5	***Nsg2***	neuron specific gene family member 2	18197
0.5	***Pnpt1***	polyribonucleotide nucleotidyltransferase 1	71701
0.5	***Ube2b***	ubiquitin-conjugating enzyme E2B	22210

The homeobox transcription factors are in green.

Supplementary Table S7. Genes with increased expression in E12.5 *Hand2*^{NC} samples.

Fold Change	Gene Symbol	Gene Title	Entrez Gene ID
73.5	***Pth***	parathyroid hormone	19226
64.0	***AA522020***	expressed sequence AA522020	103067
24.3	***Il7***	interleukin 7	16196
21.1	***1700007E05Rik***	RIKEN cDNA 1700007E05 gene	114672
21.1	***Zfp133-ps***	zinc finger protein 133, pseudogene	668917
19.7	***Hand2***	heart and neural crest derivatives expressed transcript 2	15111
19.7	***Slco1b2***	solute carrier organic anion transporter family, member 1b2	28253
14.9	***1810058N05Rik***	RIKEN cDNA 1810058N05 gene	69808
14.9	***Ighv14-2***	immunoglobulin heavy variable 14-2	668421
13.9	***Casp9***	caspase 9	12371
13.9	***Sdr16c6***	short chain dehydrogenase/reductase family 16C, member 6	242286
13.0	***Krt28***	keratin 28	70843
13.0	***Spon1***	spondin 1, (f-spondin) extracellular matrix protein	233744
12.1	***Svs5***	seminal vesicle secretory protein 5	20944
12.1	***Tnp2***	transition protein 2	21959
10.6	***4933429H19Rik***	RIKEN cDNA 4933429H19 gene	71280
9.8	***Ifna9***	interferon alpha 9	15972
9.2	***D14Ertd574e***	DNA segment, Chr 14, ERATO Doi 574, expressed	52508
9.2	***Magea1***	melanoma antigen, family A, 1	17137
9.2	***Pkd1l2***	polycystic kidney disease 1 like 2	76645
8.6	***C77370***	expressed sequence C77370	245555
8.0	***A930002I21Rik***	RIKEN cDNA A930002I21 gene	109226
8.0	***Astn2***	astrotactin 2	56079
8.0	***Cgnl1***	cingulin-like 1	68178
7.0	***Amotl1***	angiomotin-like 1	75723
7.0	***Hmx2***	H6 homeobox 2	15372
7.0	***Mcm9***	minichromosome maintenance complex component 9	71567
7.0	***Zfp819***	zinc finger protein 819	74400
6.5	***Hist1h1e***	histone cluster 1, H1e	50709
6.1	***D530015H24Rik***	RIKEN cDNA D530015H24 gene	78715
5.7	***Gpat2***	glycerol-3-phosphate acyltransferase 2, mitochondrial	215456
5.3	***Igkv1-135***	immunoglobulin kappa variable 1-135	243420
4.9	***1700003H04Rik***	RIKEN cDNA 1700003H04 gene	384775
4.9	***Smr2***	submaxillary gland androgen regulated protein 2	20600
4.6	***Samhd1***	SAM domain and HD domain, 1	56045
4.3	***Cbs***	cystathionine beta-synthase	12411
4.3	***Hoxc5***	homeobox C5	15424
3.7	***2410018L13Rik***	RIKEN cDNA 2410018L13 gene	245297

3.2	***Cx3cr1***	chemokine (C-X3-C) receptor 1	13051
3.2	***Nlrc5***	NLR family, CARD domain containing 5	434341
3.0	***AU020147***	expressed sequence AU020147	102226
2.8	***Hsf2***	heat shock factor 2	319253
2.8	***Srcap***	Snf2-related CREBBP activator protein	100043597
2.6	***1700023H06Rik***	RIKEN cDNA 1700023H06 gene	69442
2.6	***Ibsp***	integrin binding sialoprotein	15891
2.3	***2310058N22Rik***	RIKEN cDNA 2310058N22 gene	71921
2.3	***Akr1c14***	aldo-keto reductase family 1, member C14	105387
2.3	***Ccbe1***	collagen and calcium binding EGF domains 1	320924
2.3	***Gm2083***	major urinary protein LOC100048885	100038948
2.3	***Xist***	inactive X specific transcripts	213742
2.1	***Abcc9***	ATP-binding cassette, sub-family C (CFTR/MRP), member 9	20928
2.1	***Baiap3***	BAI1-associated protein 3	545192
2.1	***Hoxd4***	homeobox D4	15436
2.1	***Meg3***	maternally expressed 3	17263
2.1	***Otud5***	OTU domain containing 5	54644
2.1	***S100a8***	S100 calcium binding protein A8 (calgranulin A)	20201
2.0	***Gm13242***	predicted gene 13242	100041379
2.0	***Gsc***	goosecoid homeobox	14836
2.0	***Ngp***	neutrophilic granule protein	18054
2.0	***Slc10a6***	solute carrier family 10, member 6	75750
2.0	***Zfp386***	zinc finger protein 386 (Kruppel-like)	56220

The homeobox transcription factors are in green.

Supplementary Table S8. Dysregulated homeobox transcription factors in E12.5 *Hand2^{NC}* heads.

Gene	Protein	OMIM	Human genetic disease	Mutant mice with craniofacial phenotypes
Alx3	aristaless-like homeobox 3	*606014	Frontonasal dysplasia 1<br>CL/P	Facial cleft[9,10]
Cux1/ Cutl1	CUT-like 1	*116896	n.r.	n.r.
Gsc	goosecoid homeobox	*138890	Miller Fisher syndrome<br>Auditory neuropathy<br>short stature, auditory canal atresia, mandibular hypoplasia, skeletal abnormalities	Abnormal craniofacial morphology[11,12]
Hmx1	H6 family homeobox 1	*142992	Oculoauricular syndrome, coloboma	Cranial abnormalities[13]
Hmx2	H6 family homeobox 2	*600647	Peripartum cardiomyopathy, split foot	Abnormal inner ear morphology[14]
Hoxa4	homeobox A4	*142953	Megacolon, abdominal aortic aneurysm	n.r.
Hoxa5	homeobox A5	*142952	n.r.	Delayed ear emergence[4]
Hoxc4	homeobox C4	*142974	n.r.	n.r.
Hoxc5	homeobox C5	*142973	n.r.	n.r.
Hoxd1	homeobox D1	*142987	clubfoot, syndactyly	Fusion of atlas and occipital bones[5]
Hoxd4	homeobox D4	*142981	n.r.	n.r.
Irx5	Iroquois homeobox protein 5	*606195	Hamamy syndrome<br>sensorineural hearing loss	n.r.
Isl1	Isl LIM homeobox 1	*600366	n.r.	Abnormal pharyngeal arch morphology[15]
Lhx8	LIM homeobox gene 8	*604425	Nonsyndromic CP	CP[16]
Pax3	paired box gene 3	*606597	Waardenburg syndrome, type I<br>Waardenburg syndrome, type III<br>Craniofacial-deafness-hand syndrome<br>alveolar rhabdomyosarcoma<br>CL/P, CP	n.r.
Pax9	paired box gene 9	*167416	Tooth agenesis, selective, 3	CP, absent teeth, absent premaxilla, absent alveolar process[17,18]
Pdx1	pancreatic and duodenal homeobox1	*600733	Maturity-onset diabetes of the young, type IV<br>Pancreatic agenesis<br>Diabetes mellitus, type II,	n.r.
Phox2b	paired-like homeobox 2b	*603851	Central hypoventilation syndrome	n.r.
Pitx1	paired-like homeodomain transcription factor 1	*602149	Clubfoot, congenital, with or without deficiency of long bones and/or	CP, decrease tongue size, absent submandibular gland, small mandible[19,20]

			mirror-image polydactyly	
			Liebenberg syndrome	
			CP	
Pou3f3	Pou domain, class 3	*602480	n.r.	Absent squamosal bone and zygomatic bone, abnormal morphology of temporal styloid process, jaw, incus, and stapes[21]
Rhox4b	reproductive homeobox 4B	n.r.	n.r.	n.r.
Shox2	short stature homeobox 2	*602504	n.r.	CP[22]
Uncx	UNC homeobox	n.r.	n.r.	n.r.
Zeb2/ Zfhx1b	zinc finger E-box binding homeobox 2	*235730	Mowat-Wilson syndrome CL/P, submucous CP	Absent first pharyngeal arch[23]
Zfhx3/ Atbf1	zinc finger homeobox 3	*104155	Prostate cancer Acute myocardial infarction	n.r., complete prenatal lethality[24]

OMIM, Online Mendelian Inheritance in Man (http://omim.org); n.r., not reported; CL/P, cleft lip and/or palate; CP, cleft palate.

Supplementary Table S9. Primer sequences.

	Primer Sequence (Sense)	Primer Sequence (Antisense)
Alx3	5′-cgt gag cgt tat ggg aag at-3′	5′-aaa ggg ttt gct gtt tgg tg-3′
Atf4	5′-ctc atg ggg cct tta gga cg-3′	5′-ggt cga agg ggg aca tca ag-3′
Dner	5′-tgc gaa ctg tac aag gac cc-3′	5′-ggc cga gac ttc ttc cca aa-3′
Fli1	5′-gtc aga cca cca gac caa gg-3′	5′-cag gag agt gcc tcg tgt g-3′
Hand2	5′-cac cag cta cat cgc cta cc-3′	5′-cct tac cac acg gga gtg tc-3′
HIF1a	5′-cga gaa cga gaa gaa aaa gat gag-3′	5′-ttg atg ttc atc gtc ctc ccc-3′
Hmx1	5′-gaa gag gaa gag gac gac ga-3′	5′-gac tcg agc tgg aag acc tg-3′
Hmx1	5′-tcg atc tga agc gct acc tg-3′	5′-ctt tcc atc gga acg ggt ct-3′
Hmx2	5′-ctc cag ctt cac cat cca gt-3′	5′-tca ggt agc gtt tca tgt cg-3′
Hoxa5	5′-ggc tac aat ggc atg gat ct-3′	5′-tgg gcc acc tat att gtc gt-3′
Hoxd1	5′-cag cac ttt cga gtg gat ga-3′	5′-tat tca aag gtg ggg agc ag-3′
Irx5	5′-cac cct tat gca gca cct ct-3′	5′-tcg gag gac gac tcc tta aa-3′
Isl1	5′-tca tcc gag tgt ggt ttc aa-3′	5′-ttc cca ctt tct cca aca gg-3′
Lhx8	5′-act gca tgc tgg aca atc tg-3′	5′-ttg ttg gtg agc atc cat gt-3′
Pax3	5′-ctt ttt cgt ctc gcc ttc ac-3′	5′-aga cag cgt cct tga gca at-3′
Pax9	5′-tat tct gcg caa caa gat cg-3′	5′-ttg ggc tgg tgt agg gta ag-3′
Pbx1	5′-gca tca tcc acc gca agt tc-3′	5′-ctt cca gaa gtc agg cca cg-3′
Pcgf5	5′-acc agg atg ccc cag act at-3′	5′-tgt tct cgt agt cca ggc ac-3′
Pdx1	5′-gag ctg gca gtg atg ttg aa-3′	5′-tct ccg gct ata ccc aac tg-3′
Pitx1	5′-gtg cag agg acc cag cta ag-3′	5′-ggc atg gtc atg gaa gag at-3′
Pou3f3	5′-caa aca aaa ccg gaa gag ga-3′	5′-ttt act gcg gag gat gct tt-3′
Rb1cc1	5′-aca cta aag gaa aag cat cag ca-3′	5′-tgt ggt ctg tca taa cgt ttc tc-3′
Rhox4b	5′-cct cag gaa ctc cga ctc ag-3′	5′-tgc cac cat tgg aaa ttg ta-3′
Rsf1	5′-gtg gac act tac tgg ctt gtt aaa t-3′	5′-agc ata cat gtg gtc ttg gc-3′
Runx1	5′-cac ttc cat cct gag ctc cc-3′	5′-ttc gga agt cag cca ctg tc-3′
Sfmbt2	5′-agt gtg gct cag gca gat tt-3′	5′-tgg cta ctt agg gaa gcg tg-3′
Shox2	5′-ctg ccc cat tga tgt gtt att-3′	5′-cct cct cct cca gca cct-3′
Six4	5′-cct agt gcc cct gtg tca tt-3′	5′-gcc ttc ctg ttt cac agc tc-3′
Uncx	5′-cta ccc gga cgt gtt tat gc-3′	5′-ctt ctt tgg ctc ggg tag aa-3′
Zeb2	5′-ccc aga gag aaa ctt ggc ga-3′	5′-cct cct ggg att ggc ttg tt-3′
Zfhx3	5′-ggt gta cgg agg ctt tgt gt-3′	5′-cag gtt ctc cac atc gct ct-3′

Supplementary Figures

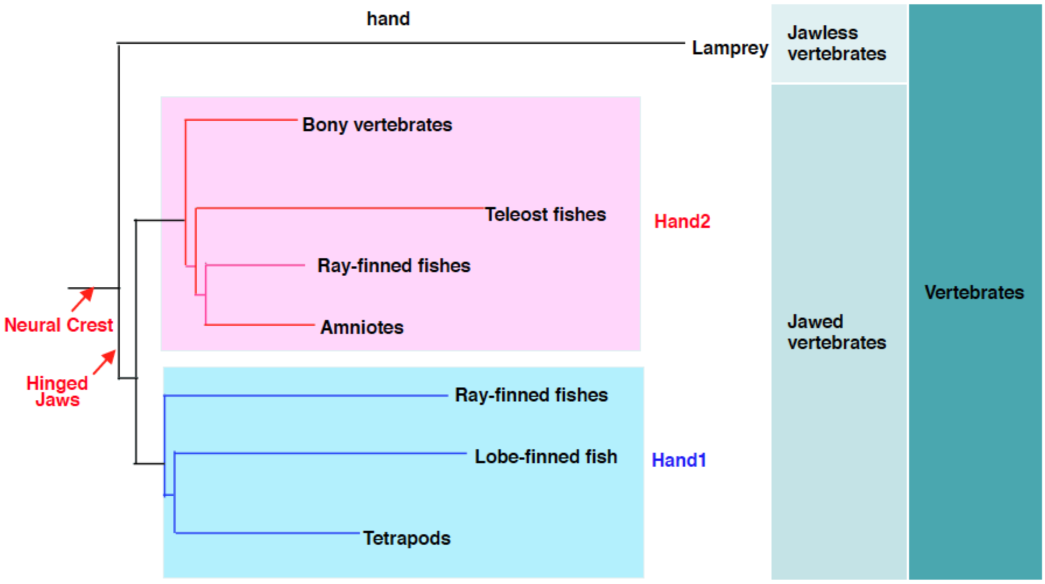

Supplementary Figure S1. Rooted phylogenetic tree of Hand proteins.

The rooted phylogenetic tree of Hand proteins was generated with Ensembl.

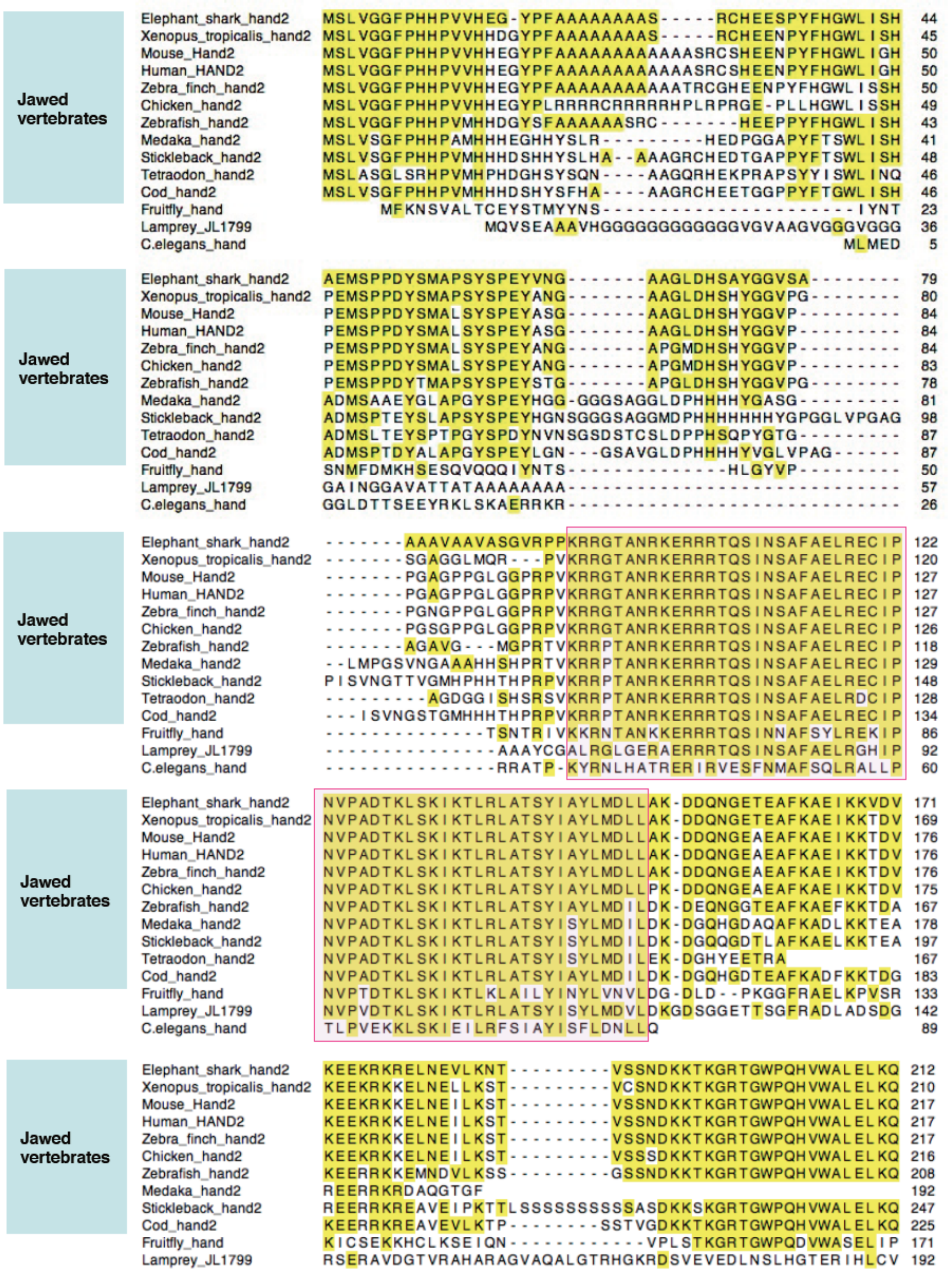

Supplementary Figure S2. Amino-acid alignment of Hand2.

The multiple sequence alignments of Hand2 orthologs were generated with ClustalW. The basic helix-loop-helix (bHLH) domain is shown with red boxes. The Hand2 amino sequences are conserved in jawed vertebrates but not in jawless vertebrates and invertebrates.

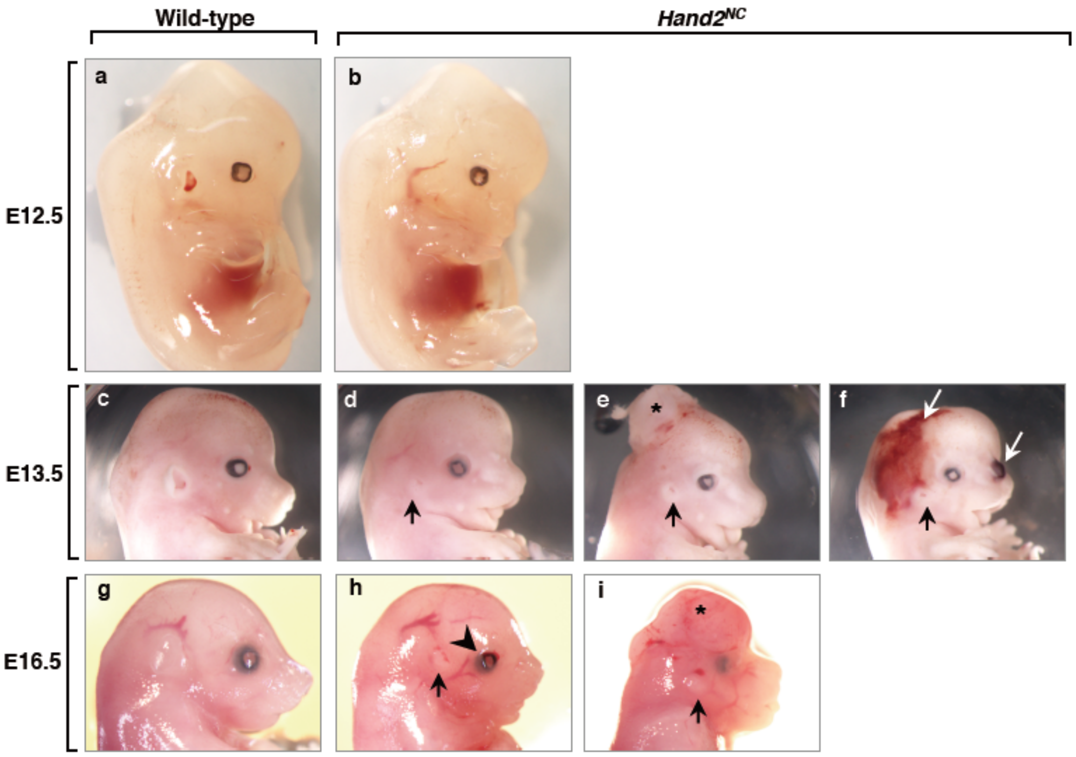

Phenotypes			
E12.5	brachycephaly, tongue protrusion		
E13.5	Inadequate pigmentation in the ventral retina, small ears, brachycephaly, tongue protrusion		
		exencephaly	hemorrhage
E16.5	open eyelids, small ears, small eye, brachycephaly, tongue protrusion		
		exencephaly	

Supplementary Figure S3. Phenotype of *Hand2^{NC}* embryos at E12.5, E13.5, and E16.5. Representative surface appearance of *Hand2^{NC}* embryos at E12.5 (a,b), E13.5 (c–f), and E16.5 (g–i). Note the small ears (black arrows), exencephaly (asterisks), and hemorrhage (white arrows) in *Hand2^{NC}* mutants at E13.5 (d,e,f) and E16.5 (h.i). An arrowhead indicates open eyelids (h).

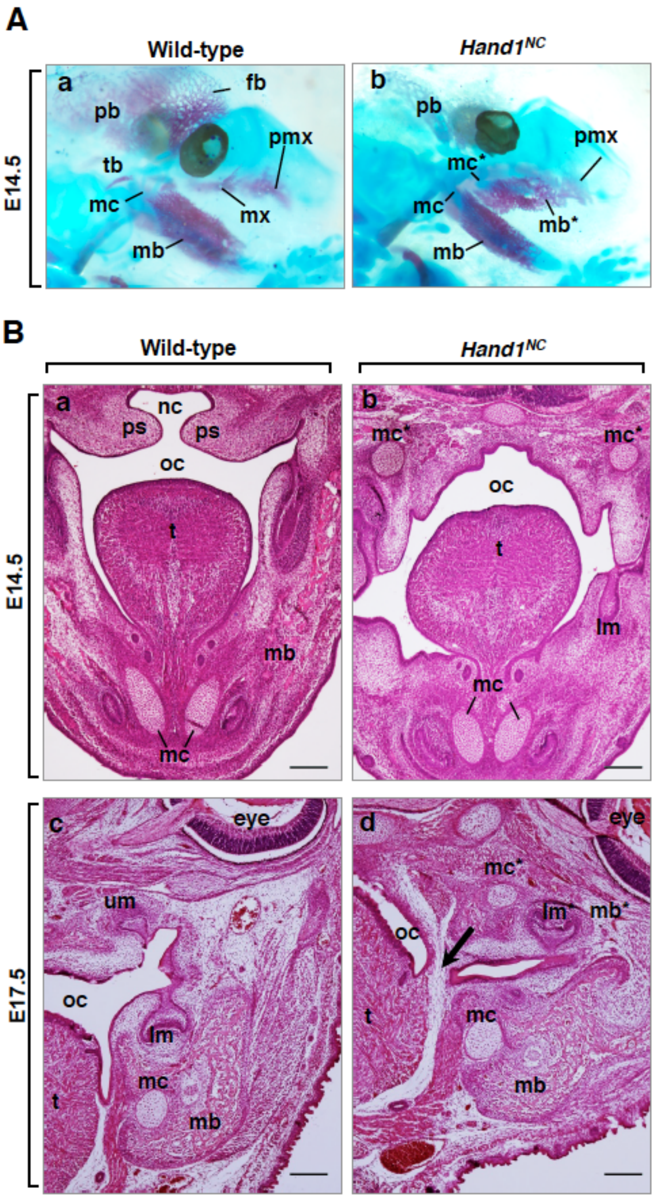

Supplementary Figure S4. Morphological transformation of the maxillary to mandibular process in *Hand1*^{NC} mutants. (**A**) Skeletal preparations from wild-type (a) and *Hand1*^{NC} (b) embryos at E14.5. The mutant maxilla (mx) is transformed to partially duplicated mandibular bone (mb*) that is associated with duplicated Meckel's cartilage (mc*). (**B**) H&E-stained coronal sections at E14.5 (a, b) and E17.5 (c,d) in wild-type (a,c) and *Hand1*^{NC} (b,d) heads. Ectopic Meckel's cartilage (mc*) and lower molar (lm*) are observed in the duplicated mandible (mb*). The original mandible of *Hand1*^{NC} mutants is fused to the duplicated mandible by the connective tissue strings (arrow in d). Scale bars: 200 µm. mb, mandibular bone; mc Meckel's cartilage; fb, frontal bone; pb, parietal bone; pmx, premaxilla; tb, temporal bone; ps, palatal shelf; oc, oral cavity; nc, nasal cavity; um, upper molar; lm, lower molar; t, tongue.

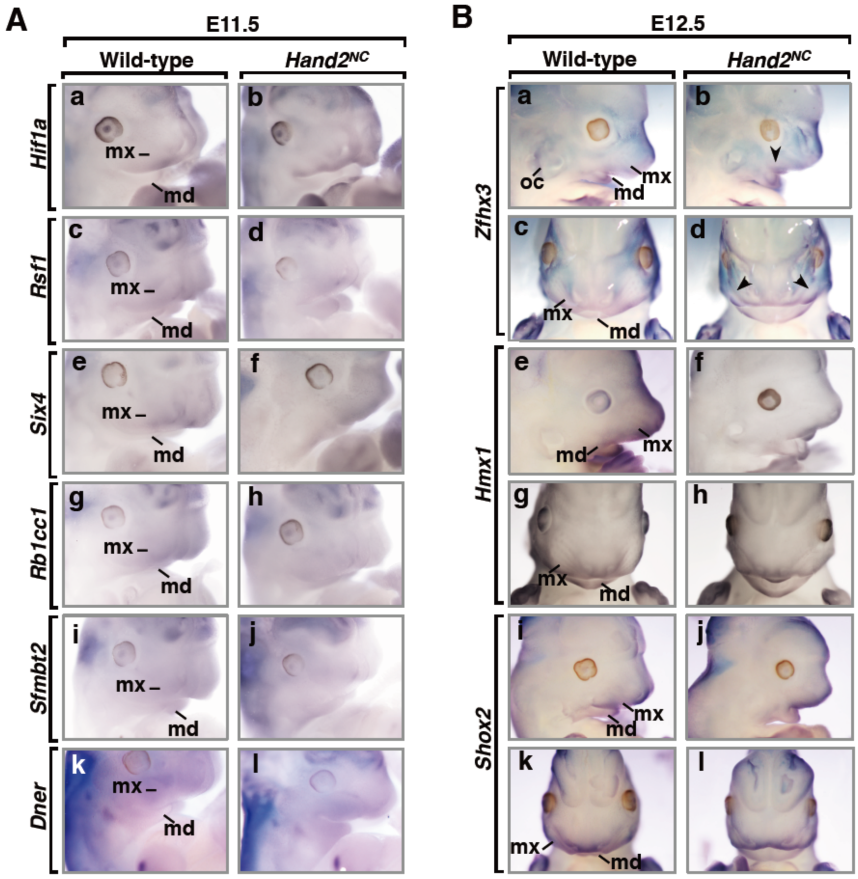

Supplementary Figure S5. Gene expression analysis of the mandibular and maxillary processes resulting from altered *Hand2* expression.

(**A**) The expression of transcription factors in the mandibular and maxillary processes at E11.5. Lateral views of wild-type and *Hand2^{NC}* embryos processed by whole-mount *in situ* hybridization, as indicated. (**B**) Expression of homeobox transcription factors in the mandibular and maxillary process at E12.5. Lateral or frontal views of wild-type and *Hand2^{NC}* embryos processed by whole-mount *in situ* hybridization, as indicated. Arrowheads indicate changes in gene expression. md, mandibular process; mx, maxillary process; oc, otic capsule.

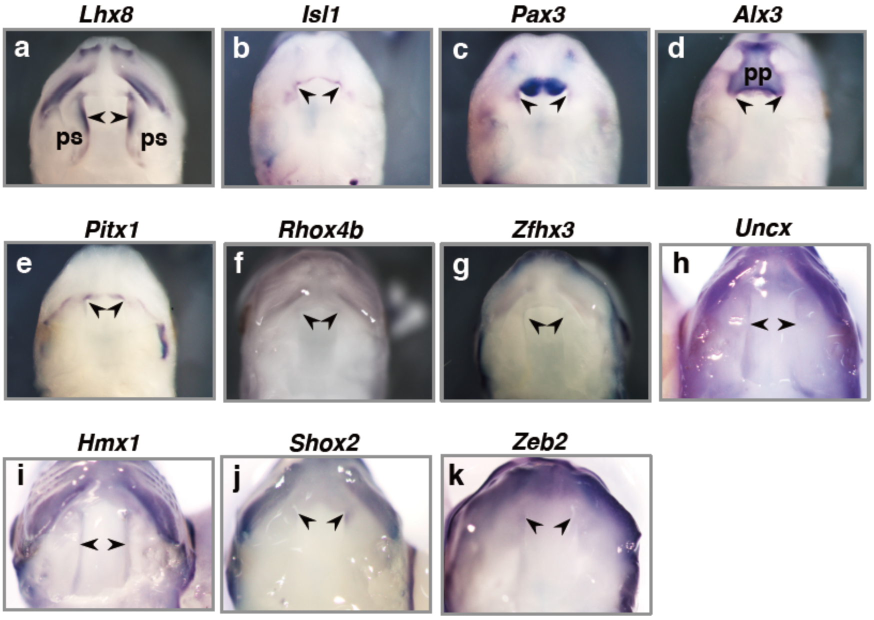

Supplementary Figure S6. Expression of homeobox transcription factors in the palatal shelves at E12.5.

Ventral views of the wild-type palatal shelves (ps) processed by whole-mount *in situ* hybridization, as indicated. Arrowheads indicate gene expression. ps, palatal shelf; pp, primary palate.

Supplementary References

1. Yang, X. et al. ATF4 is a substrate of RSK2 and an essential regulator of osteoblast biology; implication for Coffin-Lowry Syndrome. *Cell* **117**, 387-398 (2004).
2. Yanagisawa, H., Clouthier, D.E., Richardson, J.A., Charite, J. & Olson, E.N. Targeted deletion of a branchial arch-specific enhancer reveals a role of dHAND in craniofacial development. *Development* **130**, 1069-1078 (2003).
3. Iyer, N.V. et al. Cellular and developmental control of O2 homeostasis by hypoxia-inducible factor 1 alpha. *Genes Dev.* **12**, 149-162 (1998).
4. Meunier, D., Aubin, J. & Jeannotte, L. Perturbed thyroid morphology and transient hypothyroidism symptoms in Hoxa5 mutant mice. *Dev. Dyn.* **227**, 367-378 (2003).
5. Zakany, J., Kmita, M., Alarcon, P., de la Pompa, J.L. & Duboule, D. Localized and transient transcription of Hox genes suggests a link between patterning and the segmentation clock. *Cell* **106**, 207-217 (2001).
6. Selleri, L. et al. Requirement for Pbx1 in skeletal patterning and programming chondrocyte proliferation and differentiation. *Development* **128**, 3543-3557 (2001).
7. Ferretti, E. et al. A conserved Pbx-Wnt-p63-Irf6 regulatory module controls face morphogenesis by promoting epithelial apoptosis. *Dev. Cell* **21**, 627-641 (2011).
8. Ozaki, H. et al. Six4, a putative myogenin gene regulator, is not essential for mouse embryonal development. *Mol. Cell. Biol.* **21**, 3343-3350 (2001).
9. Lakhwani, S., Garcia-Sanz, P. & Vallejo, M. Alx3-deficient mice exhibit folic acid-resistant craniofacial midline and neural tube closure defects. *Dev. Biol.* **344**, 869-880 (2010).
10. Beverdam, A., Brouwer, A., Reijnen, M., Korving, J. & Meijlink, F. Severe nasal clefting and abnormal embryonic apoptosis in Alx3/Alx4 double mutant mice. *Development* **128**, 3975-3986 (2001).
11. Rivera-Perez, J.A., Mallo, M., Gendron-Maguire, M., Gridley, T. & Behringer, R.R. Goosecoid is not an essential component of the mouse gastrula organizer but is required for craniofacial and rib development. *Development* **121**, 3005-3012 (1995).
12. Yamada, G. et al. Targeted mutation of the murine goosecoid gene results in craniofacial defects and neonatal death. *Development* **121**, 2917-2922 (1995).
13. Munroe, R.J. et al. Mouse H6 Homeobox 1 (Hmx1) mutations cause cranial abnormalities and reduced body mass. *BMC Dev. Biol.* **9**, 27 (2009).
14. Wang, W., Chan, E.K., Baron, S., Van de Water, T. & Lufkin, T. Hmx2 homeobox gene control of murine vestibular morphogenesis. *Development* **128**, 5017-5029 (2001).
15. Cai, C.L. et al. Isl1 identifies a cardiac progenitor population that proliferates prior to differentiation and contributes a majority of cells to the heart. *Dev. Cell* **5**, 877-889 (2003).
16. Zhao, Y. et al. Isolated cleft palate in mice with a targeted mutation of the LIM homeobox gene lhx8. *Proc. Natl. Acad. Sci. USA* **96**, 15002-15006 (1999).
17. Peters, H., Neubuser, A., Kratochwil, K. & Balling, R. Pax9-deficient mice lack pharyngeal pouch derivatives and teeth and exhibit craniofacial and limb abnormalities. *Genes Dev.* **12**, 2735-2747 (1998).

18. Kist, R. et al. Reduction of Pax9 gene dosage in an allelic series of mouse mutants causes hypodontia and oligodontia. *Hum. Mol. Genet.* **14**, 3605-3617 (2005).
19. Szeto, D.P. et al. Role of the Bicoid-related homeodomain factor Pitx1 in specifying hindlimb morphogenesis and pituitary development. *Genes Dev.* **13**, 484-494 (1999).
20. Lanctot, C., Moreau, A., Chamberland, M., Tremblay, M.L. & Drouin, J. Hindlimb patterning and mandible development require the Ptx1 gene. *Development* **126**, 1805-1810 (1999).
21. Jeong, J. et al. Dlx genes pattern mammalian jaw primordium by regulating both lower jaw-specific and upper jaw-specific genetic programs. *Development* **135**, 2905-2916 (2008).
22. Yu, L. et al. Shox2-deficient mice exhibit a rare type of incomplete clefting of the secondary palate. *Development* **132**, 4397-4406 (2005).
23. Van de Putte, T. et al. Mice lacking ZFHX1B, the gene that codes for Smad-interacting protein-1, reveal a role for multiple neural crest cell defects in the etiology of Hirschsprung disease-mental retardation syndrome. *Am. J. Hum. Genet.* **72**, 465-470 (2003).
24. Sun, X. et al. Heterozygous deletion of Atbf1 by the Cre-loxP system in mice causes preweaning mortality. *Genesis* **50**, 819-827 (2012).
